# Enhancing *O*-linking oligosaccharyltransferase functionality through directed evolution

**DOI:** 10.1016/j.jbc.2025.110885

**Published:** 2025-11-05

**Authors:** Rachel L. Edwards, Kathleen N. McAllister, Jenna C. McGuffey, Cory J. Knoot, Anna J. Hooppaw, Joseph J. Mackel, Bibi Zhou, Lloyd S. Robinson, Nichollas E. Scott, Christian M. Harding

**Affiliations:** 1 Omniose, Saint Louis, Missouri, USA; 2 Department of Microbiology and Immunology, University of Melbourne at the Peter Doherty Institute for Infection and Immunity, Parkville, Victoria, Australia

**Keywords:** directed evolution, oligosaccharyltransferase, OTase, protein glycosylation, bioconjugation

## Abstract

Polysaccharide protein conjugate vaccines consist of bacterial polysaccharides covalently linked to carrier proteins. Bioconjugate vaccines are a type of polysaccharide protein conjugate vaccine produced by oligosaccharyltransferases, which catalyze the *en bloc* transfer of polysaccharides to specific amino acid motifs, called sequons, engineered into carrier proteins. The *O*-linking oligosaccharyltransferase PglS has been shown to have the broadest substrate repertoire, transferring virtually any saccharide to engineered carrier proteins, making it an attractive bioconjugation tool for next generation vaccine development. Yet, the efficiency of glycan transfer varies depending on the polysaccharide substrate. Successful bioconjugation hinges upon the ability of an oligosaccharyltransferase to efficiently transfer a polysaccharide to the engineered carrier protein. Therefore, enhancing glycosylation efficiency to produce carrier proteins that are highly glycosylated is a key aspect of developing scalable processes. Using directed evolution and the group B Streptococcal serotype V capsular polysaccharide as substrate, we identified single amino acid substitutions in PglS that improved enzymatic transfer of the group B Streptococcal serotype V polysaccharide to carrier proteins. Combinatorial amino acid substitutions and the incorporation of multiple sequons in the carrier protein further increased production and quality of the bioconjugate as determined by enzyme-linked immunosorbant assay and mass spectrometry (MS). Unexpectedly, the PglS variants were found to glycosylate two independent serine residues located within the sequon, a phenomenon not observed for the wildtype enzyme, resulting in significantly enhanced glycosylation activity. Such engineered PglS oligosaccharyltransferases, which increase the ratio of polysaccharide to carrier protein, are expected to improve large scale bioconjugation processes.

Polysaccharide protein conjugate vaccines, also referred to as conjugate vaccines, are among the most effective antibacterial vaccines licensed over the last 40 years (1, 2). Currently, licensed conjugate vaccines are produced almost exclusively *via* a semisynthetic approach, whereby a bacterial polysaccharide is purified from the target bacteria, chemically modified, and then covalently linked to a carrier protein (3, 4, 5). Several conjugate vaccines have been licensed and are in widespread use including those targeting *Streptococcus pneumoniae*, *Neisseria meningitidis*, *Haemophilus influenzae* type B and *Salmonella typhi* (3). While this technology has prevented countless infections since its commercialization over the past 3 decades, it has certain disadvantages including large-scale fermentation of pathogenic organisms, significant batch to batch variation, and high developability costs associated with complex chemistry, manufacturing, and control activities (6).

An alternative approach for generating polysaccharide-protein conjugates, termed bioconjugation, simplifies the process by simultaneously producing all the required components in *Escherichia coli* and subsequently linking the polysaccharide antigen to engineered carrier proteins (7). Bioconjugation requires biosynthesis of the glycan of interest (usually a bacterial surface polysaccharide like the capsule) on the lipid precursor undecaprenyl-pyrophosphate (Und-PP), a carrier protein engineered with target sequons amenable to glycosylation by the cognate oligosaccharyltransferase (OTase), and the OTase itself that can transfer fully assembled Und-PP–linked glycans *en bloc* to the sequons on the engineered carrier protein. Coexpression of these three components simultaneously within a single organism streamlines production of the polysaccharide-protein conjugate, potentially lowering manufacturing costs (1).

Bioconjugation hinges upon the ability of an OTase to transfer a glycan to the engineered carrier protein. The specificity for the glycan substrate is largely influenced by the reducing end sugar (8). Two industrially employed bacterial OTases, PglB from *Campylobacter jejuni* and PglL from *N. meningitidis*, are restricted with regards to their substrate specificity. Specifically, PglB requires an *N*-acetylhexosamine or 2,4-diamino-2,4,6-trideoxy-glucose (D-Bacillosamine) at the reducing end, whereas PglL requires either an *N*-acetylhexosamine or a galactose residue at the reducing end (8, 9). Importantly, neither enzyme appears to transfer polysaccharides with glucose at the reducing end. The recently discovered OTase PglS from *Acinetobacter baylyi* strain ADP1 has the broadest substrate repertoire known, transferring virtually any saccharide to engineered carrier proteins, including those containing glucose at the reducing end (10). Critically, three important human bacterial pathogens, *Streptococcus pneumoniae*, *Klebsiella pneumoniae*, and group B *Streptococcus* (GBS), produce capsular polysaccharides with glucose at their reducing end, making these three pathogens ideal targets for the PglS bioconjugation system (11, 12, 13). Importantly, the capsular polysaccharides of all ten known GBS serotypes (Ia, Ib, and II–IX) have a glucose at the reducing end (13, 14). A schematic of the reaction catalyzed by PglS is shown in Figure 1*A*.

**Figure 1:**
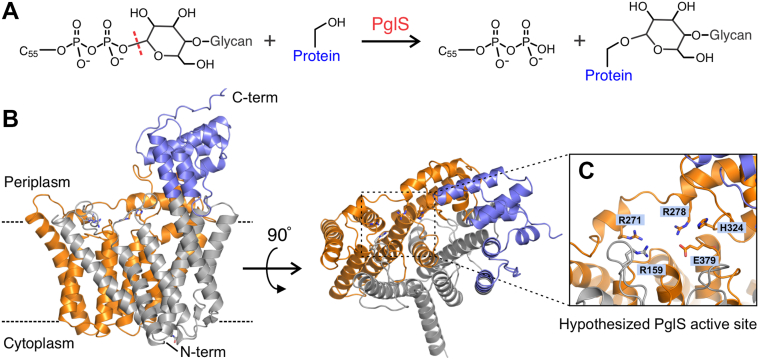
**AlphaFold model of PglS from *Acinetobacter baylyi* ADP1.** AlphaFold structure of PglS from *Acinetobacter baylyi* ADP1. *A*, schematic of the reaction catalyzed by PglS, the transfer of Und-PP–linked glycan to the hydroxyl group of a serine sidechain. C_55_ denotes the undecaprenyl lipid group. *B*, two views of the AlphaFold PglS structure with the RfaL domain colored in *orange* and the wzy_C domain colored in *blue*. The *dashed lines* in the *left panel* mark the speculated position of lipid bilayer. *C*, zoomed-in view of the putative PglS active site identified based on alignment with *C. metallidurans* WaaL (PDB ID: 7TPJ). Some key residue sidechains are shown as *sticks* and labeled. The arginine residues are proposed to aid in substrate entry and pyrophosphate coordination. H324 is hypothesized as the catalytic active site base in PglS and E379 may orient substrate for catalysis and/or participate in acid/base chemistry.

Using the PglS bioconjugation platform, we previously developed a prototype trivalent bioconjugate vaccine targeting the GBS capsular polysaccharide serotypes Ia, Ib, and III (15). Immunization of mice with the trivalent GBS bioconjugate vaccine elicited robust immunogenicity and opsonophagocytic killing (15). According to the World Health Organization, an ideal GBS vaccine should cover at least 90% of the invasive isolates for the target region (16). More than 90% of early onset disease in neonates is caused by serotypes Ia, Ib, III, and V (17). In the United States, a third of the clinical isolates are due to group B Streptococcal serotype V (GBSV), and it is the most common serotype associated with invasive disease in nonpregnant adults (18, 19, 20, 21, 22). Moreover, a significant portion of GBSV isolates are resistant to macrolide antibiotics, which complicates treatment and has driven its clonal spread because of this inherent selective advantage (23). Due to the high burden of disease caused by GBSV, this serotype will likely be included in any approved multivalent GBS vaccine. Indeed, GBSV is included in the hexavalent GBS chemical conjugate vaccine that is currently in phase II clinical trials (24, 25). Initial attempts by our group to produce GBSV bioconjugates resulted in yields insufficient for future scale-up activities. As such, we sought to improve production of GBSV bioconjugates by pursuing a directed evolution strategy to enhance PglS enzymatic activity.

In this study, we engineered PglS for improved transfer of the GBSV glycan. Selection of residues for mutagenesis was guided by an *in silico* model of PglS where key regions predicted to affect catalysis and substrate entry were specifically targeted. Saturated mutagenesis of the predicted sugar-interacting residues of PglS yielded variants with improved transfer of the glycan to engineered carrier proteins. Combinatorial amino acid substitutions, as well as incorporating a carrier protein with an enhanced number of glycosylation sites, increased production of the glycoconjugate further as determined by enzyme-linked immunosorbant assay (ELISA) and mass spectrometry (MS). Furthermore, our data indicate that these new PglS variants are broadly applicable to other saccharide substrates and are useful tools for improving bioconjugate vaccines against other global bacterial threats.

## Results

### AlphaFold model of *A. baylyi* ADP1 PglS OTase

There are currently no solved structures of any bacterial *O*-linking OTases. While smaller than the *N*-linking bacterial OTase PglB, PglS is still a relatively large protein for saturation mutagenesis, comprising 548 amino acid residues. Therefore, to focus our directed evolution screening efforts on residues hypothesized to be important for polysaccharide-protein linkage, we relied on the AlphaFold Protein Structure Database model of *A. baylyi* ADP1 PglS (Entry AF-Q6F7F9-F1-v4) to guide residue selection (Fig. 1*B*) (26). The confidence for the PglS model is reported as very high (average pLDDT 91.15). Overall, the three-dimensional AlphaFold model of PglS is similar to GT-C_B_ family of integral membrane glycosyltransferases such as WaaL and RodA which also contain 13 transmembrane helices (27, 28). While PglS has no notable features on the cytoplasmic side, the periplasmic face contains several protruding loops and a relatively large C-terminal domain (residues 438–548) that together form a cleft near the center of the protein. Based on analysis of the primary sequence using NCBI Conserved Domain Search, PglS has an RfaL O-antigen ligase superfamily domain spanning residues 163 to 396 (colored orange in Fig. 1*B*). This domain is found in the O-antigen ligase WaaL (previously known as RfaL) that catalyzes attachment of Und-PP–linked polysaccharides to the core saccharide of lipid A forming lipopolysaccharide. The RfaL domain contains many of the conserved catalytic residues and may play a role in modulating glycolipid access to the active site (27). Additionally, PglS has a Wzy_C superfamily domain spanning residues 364 to 528 (colored purple in Fig. 1*B*). Structural alignment of the *Cupriavidus metallidurans* WaaL (PDB ID: 7TPJ) and the *in silico* PglS model places the hypothesized active site of PglS in a similar location to the WaaL active site (Fig. 1*C* and Fig. S1). By directly comparing the structure of the Und-PP–bound WaaL structure (PDB ID: 7TPG) to the AlphaFold model of PglS, we hypothesized that arginine residues R159, R271, and R278 may guide substrate entry into the active site and/or coordinate the pyrophosphate moiety of Und-PP during catalysis. Similarly, structural alignment of the proposed catalytic histidine in the WaaL structure indicates that H324 of PglS may be its key catalytic residue. Residue E379 in PglS is analogous to E404 in *N. meningitidis* PglL and mutation dramatically reduced PglL activity (29). Given the similarities of the AlphaFold model of PglS to the solved cryo-EM structure of WaaL from *C*. *metallidurans* and the conserved nature of the RfaL and Wzy_C domains in PglS, these regions were selected for directed evolution. In total, we targeted 108 (20%) PglS residues to be screened. A complete list of residues selected for mutagenesis can be found in Table S1.

### Generation of *pglS* libraries with reduced codon redundancy

For our directed evolution screen, we employed the 22c-trick for saturation mutagenesis of the 108 selected amino acids (30). This method decreases the redundancy in the genetic code which in turn reduces the screening effort required for library analysis. Briefly, a mixture of three forward oligonucleotides is used in combination with a single reverse primer. The forward primers, which are designed to change a single codon, incorporate an NDT (encodes 12 unique codons), a VHG (encodes nine unique codons), and a TGG (encodes a single codon) (Table S2). The mixture contains no stop codons and only two redundancies for valine (GTT and GTG) and leucine (CTT and CTG). As a result, the redundancy is reduced to a codon to amino acid ratio of 22:20 (29). Using this PCR method, we generated 22c-trick libraries for 108 targeted amino acids of PglS. Five residues (S34, T35, Q301, T302, and F327) were unable to either be PCR amplified or recovered in sufficient quantity for library screening. As such, we used synthesized gene fragments to generate clones containing all possible amino acids at those specific sites for screening (maps displayed in Table S3).

To ensure all unique variants were represented in the library, oversampling is required. While 100% full coverage is beyond the technical limitations for large-scale random mutagenesis studies, 95% library coverage only requires 3-fold oversampling (29). Due to the minimal codon redundancy afforded by the 22c-trick method, only 66 colonies need to be analyzed for 95% coverage for a single amino acid substitution under ideal library generation conditions (30). This is in stark contrast to NNN and NNK/S saturation mutagenesis methods which, due to their high codon redundancy (64 and 32 codons, respectively), require the evaluation of at least 192 and 96 colonies, respectively, to achieve similar coverage (30). Importantly, the reduction in screening effort provided by the 22c-trick method allows for the analysis of a single amino acid library with the necessary control clones on a single 96-well plate, while maintaining 95% coverage.

### Development of a high-throughput method for screening libraries

Next, we developed a 96-well plate screening method to quantify the relative amount of GBSV polysaccharide bioconjugated to a previously established model genetically deactivated exotoxin A from *Pseudomonas aeruginosa* (EPA) carrier protein containing two PglS sequons, which is referred to as EPA2 (31). Following 22c-trick library mutagenesis, a *pglS* plasmid library was transformed into an *E. coli* W3110 mutant strain, referred to as VNM13, coexpressing the GBSV polysaccharide-lipid linked precursor. The VNM13 cell line was generated to enhance GBSV precursor production *via* three mutations. First, the *gtrABS* (also known as *yfdGHI*) glucosylating prophage system was deleted as it could potentially promiscuously modify nascent GBSV oligosaccharide repeat units. Second, the *waaL* ligase gene was deleted as the ligase and PglS compete for the same lipid-linked polysaccharide precursors. Third, the entire enterobacterial common antigen locus was deleted as this polysaccharide is also capable of being transferred to engineered carrier proteins by PglS. Deep-well plates were then inoculated with 90 randomly picked *pglS* clones from a single library, three clones of the wildtype *pglS* (pVNM245) or codon optimized *pglS* (pVNM306), and three clones of the negative control expressing a mutated PglS_H324A_ variant (pVNM322) for each library. Cultures were induced at mid-logarithmic phase and grown overnight. Periplasmic proteins were extracted *via* sucrose-lysozyme addition and added to a new set of 96-well plates precoated with purified goat anti-EPA polyclonal antibody for capture. Transfer of the GBSV polysaccharide to the EPA2 carrier protein was subsequently detected by probing with purified rabbit anti-GBSV polyclonal antibodies followed by detection with goat-anti-rabbit IgG-horseradish peroxidase.

### Identification of PglS variants with improved transfer of the GBSV glycan

**Figure 2:**
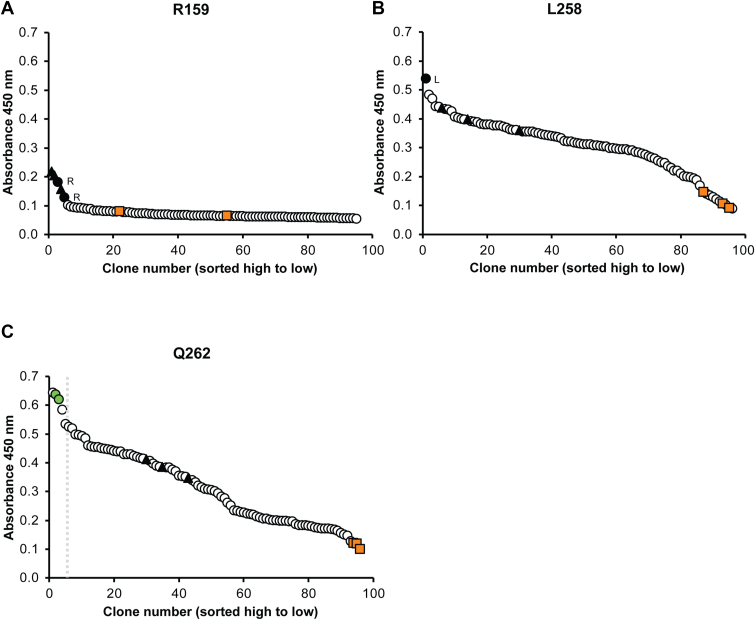
**22c library screening identifies representative amino acid substitutions that abrogate, are neutral to, or improve PglS transfer of the GBSV glycan.** Raw absorbance values for R159, L258, and Q262 library screening by capture ELISA. *Open circles* represent individual PglS library clones for R159 (*A*), L258 (*B*), and Q262 (*C*). *Black triangles* represent positive control clones expressing the WT PglS (pVNM245) sequence. *Orange squares* represent negative control clones expressing the inactive H324A PglS variant (pVNM322). *Black circles* represent clones that upon sequencing were found to have the WT (R = arginine and L = leucine) residue at the corresponding mutation site. *Green circles* represent a positive hit identified as Q262H with improved enzymatic activity as compared to the WT enzyme. The *gray dashed line* indicates a 1.4-fold cutoff compared to WT enzymatic activity. GBSV, group B Streptococcal serotype V.

In total, we screened 9720 clones (90 clones for each of the 108 amino acids targeted), by sandwich ELISA (Table S1). 22c library clones of residues R159, R271, and R278 were found to be inactive, and the only clones with absorbance values near wildtype (WT) levels were found to encode the native codon at the mutation site upon Sanger sequence analysis (black circles; Figs. 2*A* and S2, *A* and *B*). Together, these data support the model in Figure 1 suggesting that these residues may be critical for glycolipid substrate entry and/or catalysis. Most of the residues screened were amenable to substitution in that multiple amino acids could be substituted for the WT residue without significantly altering the ELISA signal (Fig. 2*B*). Importantly, we identified changes in 28 residues (26% of the libraries tested) that improved transfer of the GBSV glycan to the EPA2 carrier protein over WT levels (Table 1, Figs. 2*C* and S2, *C*–*E*). Of these residues, 15 sites could be altered to more than one amino acid. For example, the K312 residue had nine different substitutions that improved the ELISA signal when compared to the WT protein. Moreover, over 80% of the improved residues were in the predicted Wzy_C and Wzy_C_2 domains (Fig. 1).

**Table 1:** PglS screening hits

PglS residue	Amino acid substitution	Fold increase in activity over WT
N28^4^^a^	A (2), L (3), K (2), F (3)	A – 1.5, L – 1.4, K – 1.4, F – 1.8
	V (4), Y (2), W (2)	V – 1.5, Y – 1.6, **W – 2.0**
S30^3^^a^	F (5)	**F – 1.7**
N31	L (4), V (4), F (2), E (2)	L – 1.5, V – 1.4, F – 2.0, E – 1.3
S34^b^	A, V	A – 1.7, V – 1.3
T35^b^	V	1.3
W215	V (3), I (2)	V – 1.3, I – 1.3
I216^3^	G (2), V (2)	V – 1.5, **G – 1.7**
L254	W (2)	W – 1.4
L255^2,3^	G (3), C (2), V (2)	**G – 1.5**, C – 1.5, V – 1.4
F256^4^	W (4), L (3), C (2)	**W – 1.7**, L – 1.3, C – 1.4
I259^1^	V (3), L (6), M (2)	**V – 1.9**, L – 1.6, M – 1.7
Q262^1^	G (4), S (3), H (2), K (2)	G – 1.3, S – 1.5, **H – 1.7**, K – 1.3
T268^1,2^	F (5)	**F – 1.5**
F269^2,3^	W (2)	**W – 1.5**
M274	E (2)	E – 1.3
L286^3^	C (3), D (2), I (4), V (3), W (2)	C – 1.6, D – 1.3, I – 1.8, **V – 2.0**
		W – 1.4
E280^1^	L (2)	**L – 1.4**
K287^4^	H (2), M (4), P (2)	H – 1.6, **M – 2.2**, P – 1.6
H291^2,3^	R (7)	**R – 1.5**
Y297^2,4^	W (3)	**W – 1.8**
G300^2,4^	L (6), Q (2), V (3)	**L – 1.5**, Q – 1.5, V – 1.5
K312^1,2^	W (4), S (4), Q (4), E (9), G (10), N (2), A (4), L (2), V (3)	W – 1.3, S – 1.6, Q – 1.4, **E – 1.8**
		G – 1.4, N – 1.5, A – 1.4, L – 1.3, V – 1.3
S313	D (2)	D – 1.4
T315^2,3^	A (3), V (2)	**A – 1.6**, V – 1.5
N325^3,4^	A (5)	**A – 1.5**
M331^4^	C (3)	**C – 1.6**
L378^1,4^	T (2), V (2)	T – 1.4, **V – 1.5**
P381^1^	L (2)	**L – 1.5**

Hits identified *via* ELISA library screening. Values in parentheses indicate the number of times that amino acid was observed on a single ELISA plate. Fold increase in OTase activity is the mean fold change over the WT values observed in the ELISA. Residues in bold were selected for combinatorial screening. 1-4 indicates on which combinatorial screening plate the residue was included.

a Denotes residues that were incorporated into combinatorial backbones.

b Indicates residues that were identified through targeted substitutions.

### Combinatorial analysis of PglS hits identify variants with enhanced glycan transfer

**Figure 3:**
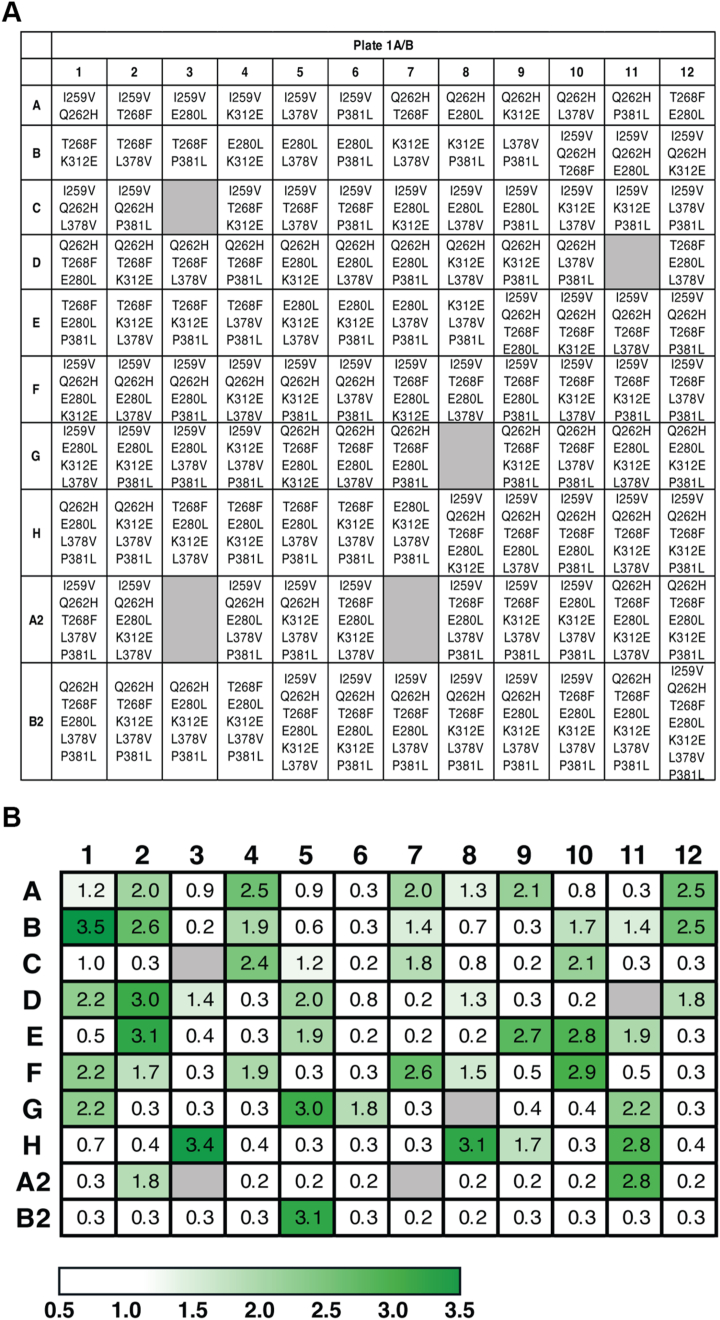
**Combinatorial analysis of the PglS hits identifies additive amino acid substitutions that improve GBSV glycan transfer more than a single amino acid substitution.***A*, plate 1A/B map depicting the combinations of substitutions analyzed by sandwich ELISA. *Gray* indicates empty wells. *B*, heat map of the fold changes in ELISA signals for the combinatorial substitutions compared to WT PglS as explained in the color key. Wells correspond to the combinations listed in *panel A*. The fold changes represent the mean from at least two independent experiments. GBSV, group B Streptococcal serotype V.

To further improve PglS activity, we employed a combinatorial approach using the hits identified in Table 1. Given the large number of hits identified (28 hits), a full factorial analysis of all hits was both time and cost prohibitive. As such, we narrowed our combinatorial analysis using the following criteria. First, we selected hits with at least a 1.4-fold improvement in ELISA signal over WT levels. Second, we prioritized substitutions most frequently observed and those that had the highest fold change (FC). A complete list of all combinations tested can be found in Fig. S3 and Table S3. Next, gene fragments containing the different combinations were synthesized as dsDNA fragments, cloned into the *pglS* backbone, electroporated into our VNM13 GBSV expression strain, and analyzed *via* sandwich ELISA as described above. Each 96-well screening plate contained combinatorial iterations of hits starting with at least two amino acid substitutions and going up to either seven or eight substitutions depending on the plate. A total of 443 combinations were evaluated (Figs. 3*A*, S3, and Table S3).

The mean FCs for the single PglS substitutions incorporated in plate 1A and plate 1B ranged from a 1.4- to 1.9-fold increase in ELISA signal compared to WT PglS (Table 1). However, screening the combinatorial plates indicated that 27 sets of substitutions (23% of combinations tested) had a signal increase of at least 2-fold, with seven sets having 3-fold or greater ELISA values than WT PglS (Fig. 3). Importantly, well B1 which contained the PglS_T268F K312E_ double amino acid substitution displayed a 3.5-fold increase in signal. This is in comparison to the single amino acid changes which have a 1.5- and 1.8-fold increase, respectively (Table 1), and suggests that there is an additive effect with these two amino acid changes. Additional substitutions to PglS_T268F K312E_ did not further increase the ELISA signal, and in many cases caused a decrease in ELISA signal (compare well B1 with wells C4, D2, E2, and E3) (Fig. 3). Despite P381L showing a 1.5-fold increase in ELISA signal as a single amino acid change (Table 1), its incorporation into all combinatorial sets proved inhibitory (Fig. 3), and was therefore removed in further rounds of screening.

Additional sets of combinatorial PglS substitutions were subsequently analyzed (Fig. S3). More than half of the sets (57%) on plate 2 had a FC ≥3.0, and of these, 26 contained PglS_T268F K312E_ in the final combination. However, none of the wells had an ELISA signal that was higher than the 3.5-FC displayed by the double variant alone (Fig. 3).

Since several wells that had FCs ≥3.0 did not incorporate either T268F or K312E substitutions into PglS, we asked whether other combinations could have larger effects on the transfer of the GBSV glycan. During 22c library screening, we identified five residues near the N terminus with increased OTase activity (Table 1). However, due to technical limitations, these residues could not be evaluated in conjunction with hits identified in the Wzy_C and Wzy_C_2 domains, as the distance between the amino acids of interest was too great for eBlock synthesis by Integrated DNA Technologies (IDT) at the time. To circumvent these constraints, we first constructed N28W or S30F amino acid changes in PglS and then analyzed combinatorial substitutions applied in either of these backgrounds. Plates 3A and 3B, combinations analyzed in the S30F background, showed substantially lower ELISA signals throughout, with only four wells having an ELISA signal more than 2-fold over WT PglS. This suggests that either the S30F incorporation was deleterious, or the combinations of Wzy_C and Wzy_C_2 amino acid changes analyzed were suboptimal (Fig. S3, *C* and *D*). A handful of combinations on plates 4A and 4B, combinations analyzed in the N28W background, displayed higher ELISA signals than WT PglS, with six combinatorial substitutions displaying FCs ≥3.0 (Fig. S3, *E* and *F*). Nevertheless, none of these sets achieved higher OTase activity than the PglS_T268F K312E_ variant identified on plates 1A and 1B (Fig. 3). Accordingly, the PglS_T268F K312E_ double variant was selected as a lead candidate for further testing. However, since the combinations screened in the N28W background did not contain either T268F or K312E, we generated the PglS_N28W T268F K312E_ triple variant and included it in addition to the PglS_T268F K312E_ double variant in all subsequent experiments.

### Phenotypic characterization of the engineered variants indicates improved transfer of the GBSV glycan

The EPA2 carrier protein was used as the model carrier protein for the 22c-trick library screening as well as the combinatorial hit screening. However, a derivative of the EPA2 protein, referred to as EPA6, is the preferred carrier protein for GBS bioconjugate vaccine development as it has six sequons strategically placed around surface-exposed loops of EPA to maximize antigen presentation. A manuscript detailing the development of EPA6 as an optimal carrier protein is in final stages of revision for publication. Since the EPA6 carrier protein has the potential for an improved polysaccharide to protein ratio when compared to EPA2, it was used moving forward to determine if enhanced PglS enzymatic activity also translated to the modified EPA6 carrier protein or if the evolution process was specific to EPA2.

**Figure 4:**
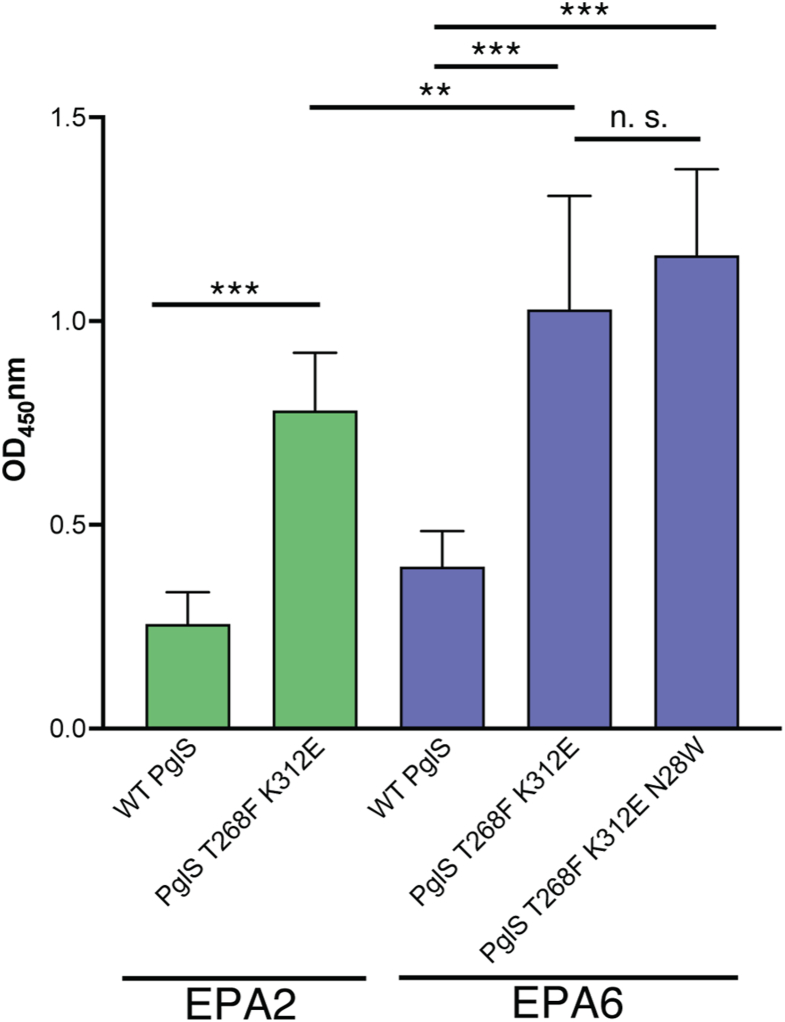
**Evolved PglS variants have improved OTase activity for the GBSV glycan.** ELISA values indicating transfer of the GBSV glycan by the PglS variants to EPA2 (*green*) or EPA6 (*purple*) carrier proteins. The means ± SD from at least three independent experiments are displayed (one-way ANOVA, ∗∗*p* < 0.001, ∗∗∗*p* < 0.0001).

The efficiency of the WT PglS and the PglS variant OTases to transfer the GBSV glycan to the EPA2 and EPA6 carrier proteins was compared by ELISA. Like our combinatorial analysis (Fig. 3*B*), data indicate that transfer of the GBSV glycan to the EPA2 carrier protein was significantly improved for the PglS_T268F K312E_ variant when compared to the WT PglS (Fig. 4 green bars, *p* < 0.0001; Fig. S4 green bars FC = 2.8). This effect was observed for glycan transfer by the double amino acid substitution to the EPA6 carrier protein as well, with a 2.7-fold (Fig. S4) improvement in transfer when compared to WT PglS enzyme (Fig. 4; purple bars *p* < 0.0001). While glycan transfer by WT PglS to EPA2 *versus* EPA6 was not statistically significant, the PglS_T268F K312E_ double variant had increased transfer to EPA6 compared to the EPA2 carrier protein (Fig. 4, *p* < 0.001). Comparable glycosylation of EPA2 and EPA6 carriers is consistent with PglS activity, not sequon availability, limiting transfer of GBSV glycan. Importantly, the incorporation of the N28W in the PglS triple substitution further improved GBSV glycan transfer to EPA6 (Fig. 4, purple bars *p* < 0.0001; Fig. S4, FC = 3.3). Taken together, these data suggest that the PglS_N28W T268F K312E_ variant had enhanced OTase activity and that the additional sequons in EPA6 in combination with the evolved PglS may further increase yields of purified bioconjugates, including GBSV bioconjugates.

### The PglS_T268F K312E_ and PglS_N28W T268F K312E_ substitutions are more efficient than WT PglS variant and promiscuously glycosylate a second serine within its sequon

We sought to further investigate the mechanism behind the enhanced glycosylation phenotype observed by the double (PglS_T268F K312E_) and triple (PglS_N28W T268F K312E_) variants as compared to WT PglS. For these studies, we developed a simplified glycosylation system using a new mutant *E. coli* W3110 strain, whereby, the only putative lipid-linked carbohydrate available for transfer by PglS or a variant thereof was a single GlcNAc residue. The W3110 genome endogenously contains a transposon interrupting *wbbL*, the rhamnosyltransferase responsible for transferring the rhamnose residue to the reducing end GlcNAc of the native O16 antigen. This transposon renders W3110 incapable of making the O16 antigen and prevents downstream glycosyltransferases in the O16 cluster from adding subsequent monosaccharides. The majority of the enterobacterial common antigen cluster was also deleted except for *wecA* as the WecA protein transfers a phospho-GlcNAc (GlcNAc-P) to undecaprenyl-phosphate (P-Und) generating a pool of GlcNAc-PP-Und available for PglS. We again deleted the glucosylating prophage cluster *gtrABS* for reasons explained above. This strain, referred to as VNM45, was then transformed with an EPA6 bioconjugation plasmid expressing either the WT, the double variant, or the triple variant of PglS. Transformants were scaled to multiliter capacity, induced for bioconjugation, and GlcNAc-EPA6 glycoproteins were purified from each background.

**Figure 5:**
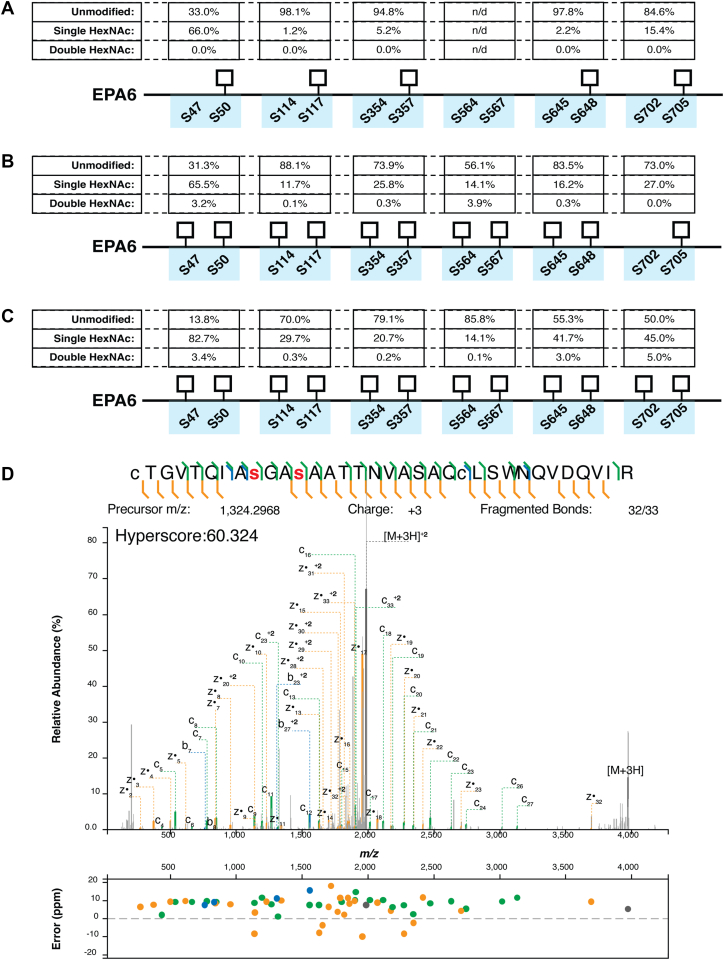
**Tandem mass spectrometryanalysis of glycopeptides extracted from cells expressing WT PglS, the double PglS variant, or triple PglS variant.** Schematic of the relative abundancies of the unmodified, singly glycosylated, and doubly glycosylated peptides. Relative peptide abundancies from GlcNAc-EPA6 tryptic peptides extracted from cells expressing (*A*) WT PglS, (*B*) PglS_T268F K312E_, and (*C*) PglS _N28W T268F K312E_. *D*, EThcD spectra of double glycosylated sequon containing glycopeptide CTGVTQIASGASAATTNVASAQCLSWNQVDQVIR for cells expressing the PglS _N28W T268F K312E_ variant. EPA, exotoxin A from *Pseudomonas aeruginosa;* EThcD, electron-transfer/higher-energy collision dissociation.

Purified EPA6 extracted from cells expressing the WT PglS was subjected to trypsin digestion allowing the identification of glycosylation events and quantification of relative glycosylation levels. Glycosylation within five of the six sequons of EPA were identified with glycosylation events localized using MS electron-transfer/higher-energy collision dissociation (EThcD) confirming Serine 12 of the PglS sequon previously defined by our group (C^1^T^2^G^3^V^4^T^5^Q^6^I^7^A^8^S^9^G^10^A^11^S^12^A^13^A^14^T^15^T^16^N^17^V^18^A^19^S^20^A^21^Q^22^C^23^) was the preferred site of glycosylation within each sequon. Furthermore, assessment of the relative abundances of glycosylated and nonglycosylated forms of peptides based on extracted ion chromatograms demonstrated variable occupancy from sequon to sequon and no evidence of multiply glycosylated peptides (Fig. 5*A* and Table S4).

Assessment of glycosylation of EPA6 purified from strains expressing either the double (PglS_T268F K312E_) or triple (PglS_N28W T268F K312E_) PglS variant confirmed glycosylation of all six sequons (Fig. 5*B*, Table S5 and Fig. 5*C*, and Table S6, respectively), which was not the case for the WT variant. Unexpectedly, we observed additional glycopeptides that were doubly glycosylated, containing two HexNAc residues per tryptic peptide/sequon. Localization data were examined and confirmed that in addition to Serine 12, a second serine, Serine 9, of the PglS sequon (C^1^T^2^G^3^V^4^T^5^Q^6^I^7^A^8^S^9^G^10^A^11^S^12^A^13^A^14^T^15^T^16^N^17^V^18^A^19^S^20^A^21^Q^22^C^23^) was also found be glycosylated (Fig. 5*D*). In addition, relative abundances of the glycopeptides and unmodified peptides were determined and showed enhanced glycosylation efficiency for the singly glycosylated glycoforms as compared to the singly glycosylated GlcNAc-EPA6 extracted from cells expressing WT PglS (Fig. 5). Collectively, we observed that the engineered PglS variants not only more efficiently glycosylate Serine 12 of the native sequon but also promiscuously glycosylate a second serine, Serine 9, of the sequon (Fig. 5). Importantly, the enhanced glycosylation efficiency of associated with the PglS_T268F K312E_ and PglS_N28W T268F K312E_ variants was not due to increased expression of EPA6 as Western blotting of whole-cell lysates comparing multiple clones demonstrated equivalent protein expression (Fig. S5). We also probed for PglS expression by adding a C-terminal FLAG tag to the different PglS variants; however, no anti-FLAG signal could be detected for any variant when analyzed *via* Western blotting. Western blotting for PglS-FLAG signal was performed on whole-cell lysates, ultracentrifuged and detergent solubilized membranes, as well as sonicated and detergent solubilized lysates.

## Discussion

Here, we engineered PglS for enhanced transfer efficiency of the GBSV glycan to a modified EPA carrier protein containing two or six sequons. Structural insights from our *in silico* model identified key regions in PglS that may be critical for substrate entry and catalysis (Fig. 1). Using a 22c-trick methodology for targeted mutagenesis, we identified 28 PglS variants with significantly improved OTase activity for the GBSV glycan when compared to WT PglS as determined by ELISA (Table 1). Additional refinements, including analysis of 443 combinatorial amino acid substitutions and incorporation of an EPA carrier protein with six sequons, further boosted bioconjugate production in our assay (Figs. 3, 4, S3, and S4). Most importantly, MS determined that *E. coli* containing either the double PglS_T268F K312E_ or the triple PglS_N28W T268F K312E_ variant could multiply glycosylate each sequon in EPA6 (Fig. 5), an outcome entirely unexpected when we first sought to enhance enzymatic activity. We predict that our enhanced PglS variants may be broadly applicable to other OTase polysaccharide substrates, offering a powerful tool for improving glycoconjugate vaccine production.

Our directed mutagenesis approach identified a suite of PglS residues that increased GBSV glycan transfer, which was further augmented by combining the identified hits (Table 1, Fig. 3 and Supporting Information S3). It is likely that more improvements can be accomplished by additional combinatorial screening; however, due to cost and the technical limitations of our medium-throughput approach, this was outside of the scope of practicality for this study. It is possible that transfer could be amplified further by incorporating additional PglS amino acid substitutions. Indeed, data indicate that a combination of 16 substitutions in the PglB OTase from *C. jejuni* increased transfer of *S. pneumoniae* serotype 8 glycan to EPA 5040-fold (WO 2021/028303 A1; PCT/EP2020/072102). Future studies should explore whether additional combinations of amino acid changes in PglS could further improve transfer efficiency to modified EPA carrier proteins. More intriguing is the possibility of applying machine learning to guide directed evolution for exponential jumps in enzymatic activity; however, the absence of a complete structural characterization of an *O*-linking OTase in complex with a glycolipid and peptide ligand complicates such modern approaches.

Mapping the N28, T268, and K312 residues onto the AlphaFold structure shows that these residues are all roughly 20 to 21 Å from the active site as measured to the imidazole sidechain of H324 (Fig. S6*A*). This positioning is surprising given the pronounced effect they have on PglS activity, allowing the OTase to glycosylate protein substrate at both a higher rate and at more sites. We speculate that these residues could influence PglS conformational changes that are required for catalysis. Limited proteolysis experiments with the related OTase PglL suggested that lipid-linked saccharide binding triggers a conformational change in the OTase (29). Similarly, molecular dynamic simulations of the O-antigen ligase WaaL showed that a transmembrane helix in the RfaL domain may switch between an open and closed state to admit Und-PP substrate entry to the active site (27). Residue T268 is positioned immediately adjacent to the analogous helix in the AlphaFold PglS model (Fig. S6*B*) and replacement of threonine with the bulkier phenylalanine may alter protein dynamics allowing for faster substrate entry or Und-PP egress. Similarly, the change in ionic charge of K312 from positive to negative in D312 could affect PglS dynamics during Und-PP and/or protein binding. Residue N28 is on the opposite side of PglS when compared to T268 and K312, specifically, positioned near the analogous site of lipid A binding in WaaL (27). PglS does not use lipid A as a substrate, but this side of the enzyme may play a conserved role in recognizing protein substrate and/or interacting with the Sec translocon to glycosylate protein prior to folding in the periplasm (32, 33). Changing N28 to the larger W28 would likely affect these processes.

To enhance bioconjugation platforms for glycoconjugate vaccine development, both polysaccharide-precursor biosynthesis and glycosylation of the engineered carrier protein itself may be optimized for efficiency and scalability. Multiple approaches are currently being applied to boost polysaccharide precursor production, mainly through mutagenesis of endogenous *E. coli* pathways that may compete for metabolic precursors or through recombinant overexpression of essential components to boost the target polysaccharide biosynthesis process. Conversely, very few approaches have been developed to enhance enzymatic activity of OTases, with no publicly known improvements made to any *O-*linking OTase system date. One approach for enhancing glycosylation relies on screening optimal sites for sequon integration. Indeed, we recently demonstrated specific sites within the EPA protein are optimal for recognition and/or glycosylation by PglS (manuscript in final stages of revision). These sequon engineering strategies can further fine-tune the glycosylation pattern to potentially elicit a superior immune response by promoting multiantennary-like presentation of polysaccharide antigens in an array pattern more natural to the bacterial presentation. In this study, we employed an EPA carrier protein with two or six sequons strategically placed around the exposed surface to amplify antigen presentation. Surprisingly, when used in conjunction with the PglS_T268F K312E_ and PglS_N28W T268F K312E_ variants, we observed both monoglycosylation and diglycosylation of the carrier protein, with slightly higher serine glycosylation detected with the triple variant for most residues (Fig. 5). In contrast, double glycosylation of EPA6 was not observed with WT PglS (Fig. 5). By incorporating additional glycosylation sites into the carrier protein, and by shifting the flux towards more polyglycosylation *via* the use of the double and triple PglS variants, our new bioconjugation system produces superior polysaccharide to protein mass ratios, which may further enhance immunogenicity. Furthermore, since conjugate vaccines are dosed on polysaccharide concentration rather than protein, this improved saccharide to protein ratio directly impacts scalability. Finally, preliminary data indicate that our enhanced bioconjugation platform is applicable to the other nine GBS serotypes, thereby boosting the commercial potential for a decavalent GBS vaccine (unpublished data).

OTases exhibit distinct sequon specificities that determine their efficiency and the range of protein substrates amenable to glycosylation. In eukaryotes, the *N-*linking OTase requires a constrained N-X-S/T motif (34, 35). In contrast, the PglB enzyme from *C. jejuni* recognizes the D/E-X-N-X-S/T sequon, where X refers to any amino acid except proline (36). The relaxed specificity of PglB enables glycosylation of a wider range of proteins, making it a valuable tool for glycoengineering (37). For PglS, data indicate that an 11-amino acid sequence (-IASGASAATTN-) derived from the carrier protein ComP constitutes the minimal sequon required for glycosylation (31). This broader specificity makes PglS an attractive glycoengineering tool as it can glycosylate proteins beyond the constraints displayed by PglB. Due to its efficient function in recombinant systems and its uniquely promiscuous glycosylation of sequons, we anticipate that the PglS enzyme will offer new opportunities for customizable glycosylation in biotechnology.

## Experimental procedures

### Bacterial strains and plasmids

The GBSV capsule locus was cloned into the vector pBBR1MCS2 using a Gibson assembly strategy (New England BioLabs) using the reference strain CBJ111 serotype V genomic DNA as template. Briefly, the capsule cluster was PCR-amplified using primers aaaagctgggtaccgggccccccctcgaggATGATTCAAACAGTTGTGGTTTATTTTTC and attcgatatcaagcttatcgataccgtcgaTTATAAGGTTTTAACTTCGTCTACAAATAATTG. Each primer contained a 20 bp homology arm to the multiple cloning site of pBBR1MCS2 and the vector linearized *via* PCR using the 5′ primer: tcgacggtatcgataagcttg and the 3′ primer: cctcgagggggggcc. The PCR products were gel-purified, used for the Gibson assembly reaction, and then transformed into DH10b cells. Transformants were selected on LB agar supplemented with 20 μg/ml kanamycin (Gold Biotechnology #K-120-50). Plasmid DNA was verified by Sanger sequencing (Genewiz).

To maximize the amount of glycan substrate available for PglS, *waaL*, *gtrABS* (*yfdGHI*), and the enterobacterial common antigen gene cluster (*rfe - yifK*) were sequentially deleted from *E. coli* W3110 using lambda RED recombinase-mediated recombination (38). For each deletion, the expected chromosomal alteration was confirmed by PCR with flanking primers, and the antibiotic resistance cassette was excised using the flippase recombinase recombinase encoded by pCP20. This final strain was designated VNM13.

VNM13 cells were made electrocompetent by growing cells to the mid-logarithmic stage followed by two rounds of washing in 10% glycerol and a final resuspension in 1/1000 of the original culture volume. Electrocompetent VNM13 cells were then electroporated with 1.5 μl pBBR1MCS2-GBSV in a chilled cuvette and cells allowed to recover for 1 h at 37 °C in 500 μl Super Optimal broth with Catabolite repression (Invitrogen #15544-034). Bacteria were plated on LB agar supplemented with 20 μg/ml kanamycin. VNM13 cells containing pBBR1MCS2-GBSV were subsequently made electrocompetent as described above and then used for *pglS* library screening.

To add the N28W substitution into the PglS_T268F K312E_ variant, primer pair pVNM306_N28W_Fwd/pVNM306_N28W_Rev was used to amplify pVNM432 before treatment with *DpnI* at 37 °C for 2 h to remove template DNA (Table S2). Gel purified pVNM432_N28W was then assembled using NEBuilder HiFi DNA Assembly master mix as described in the manufacturer’s protocol. Two microliters of the HiFi reaction was transformed into 50 μl of chemically competent Stellar competent cells (Takara Bio #ST0213). Transformants were selected on LB supplemented with 100 μg/ml ampicillin. Clones were verified using Plasmidsaurus whole plasmid sequencing to verify presence of the N28W substitution. This resulting construct was designated pVNM457.

To potentially detect PglS protein expression, a FLAG tag (amino acid sequence DYKDDDDK) was fused at C terminus of PglS in the plasmids pVNM432, pVNM457, and pVNM599 using primer BZ058 (ggaggcggtagcgattataaagacgatgatgacaaatgacctgcaggcatgcaagc) and primer BZ059 (cgtctttataatcgctaccgcctccgtactcaatggagcaaaacttcaacttgggc), generating a linearized vector containing the C-terminal FLAG tag operably fused to *pglS*. The PCR product was gel purified and used for self-assembling HiFi reaction to recombinantly fuse the plasmid back together. HiFi reactions were transformed into Stella competent cells, transformants were selected on LB supplemented with 100 μg/ml ampicillin. Clones were verified using Plasmidsaurus whole plasmid sequencing to verify the presence of FLAG tag generating the new plasmids pVNM771, pVNM772, and pVNM773, respectively.

### Purification of goat anti-EPA and rabbit anti-GBSV antibodies

Goat polyclonal anti-EPA antibodies (Biosynth Cat. 70-1006) were purified using a 1 ml HiTrap Protein G HP column (Cytiva) on an ÄKTA pure 25 L fast protein liquid chromatography (FPLC) instrument (Cytiva). Three vials of lyophilized antisera were diluted in 20 ml PBS buffer and filtered through 0.45 μm PES syringe filter (Millex-GP) before loading on the column. The column was equilibrated in 20 mM sodium phosphate buffer pH 7.0 (buffer A). All chromatography was performed at a 1 ml/min flow rate. Sera were loaded on the column and washed with ten column volumes buffer A. Antibodies were eluted using five column volumes 0.1 M glycine-HCl pH 2.7 (buffer B) and 0.5 ml fractions were collected in 96-well plates (Greiner Bio-One). Glycine-HCl was neutralized with 50 μl 1 M Tris–HCl pH 9.0 in each well. The purity of the antibodies was assessed using SDS-PAGE and Coomassie gel staining. Fractions containing the purified antibodies were pooled and diluted to 1 mg/ml in PBS prior to use. Rabbit polyclonal anti-GBSV sera (SSI Diagnostica Ref. 22461) were purified using a 1 ml HiTrap Protein A HP column (Cytiva) and Äkta pure 25 L FPLC instrument. Two vials (2 ml) were diluted in 28 ml PBS buffer and filtered through a 0.45 μm PES syringe filter. For purification, the same FPLC methods were used as for the protein G column except buffer B contained 0.1 M citric acid pH 3 and each plate well contained 175 μl 1 M Tris–HCl pH 9.0. The purified antibodies were stored at −80 °C prior to use.

### Mutagenesis of *pglS* using a 22c library approach

Site-saturation mutagenesis libraries were generated using the 22c-trick method (30). For each mutation site, four primers were used—3 forward and 1 reverse (Table S2) (IDT). The three forward primers NDT, VHG, and TGG (where H = A/C/T) were used at a 12:9:1 ratio. This eliminates stop codons and generates an almost equal distribution of amino acids: 2/22 for leucine and valine and 1/22 for the remaining 18 amino acids. Primers were diluted in water to a final working concentration of 10 μM. PCR was carried out using the Phusion High Fidelity PCR Master Mix according to manufacturer’s instructions (New England BioLabs #F-531). Products were purified with the GeneJET PCR Purification Kit (Thermo Fisher Scientific #K0702) and eluted in 44 μl water. The template DNA was degraded by adding 1 μl *DpnI* and 5 μl rCutSmart buffer to the eluate and incubating for 2 h at 37 °C (New England BioLabs #R0176L). Digested PCR products were separated by gel electrophoresis on 1% agarose (Gold Biotechnology #A-201-500), and the products extracted using GeneJET Gel Extraction Kit (Thermo Fisher Scientific #K0692). PCR products were combined 1:1 with the HiFi DNA Assembly Master Mix (New England BioLabs #E2621X) and ligated for 1 h at 50 °C. For *E. coli* transformation, 2 μl of the ligation reaction was added to 40 μl Stellar competent cells, and the reaction chilled on ice for 30 min. The cells were heat shocked at 42 °C for 50 s and then chilled on ice for 2 min. Bacteria were recovered in 500 μl Super Optimal broth with Catabolite repression (Takara Bio #636763) while shaking for 1 h at 37 °C. Cells were plated on LB agar containing 100 μg/ml ampicillin (Gold Biotechnology #A-301-100) and incubated overnight at 37 °C. To ensure full library coverage, 300 to 500 colonies from each transformation were combined and washed with 10 ml 1X PBS. Cells were pelleted by centrifugation at 4000*g* and resuspended in 500 μl resuspension buffer (GeneJET Plasmid Miniprep Kit, Thermo Fisher Scientific #K0503). The resuspended cells were then divided in half and miniprepped per the manufacturer’s instructions. To verify the residues were successfully mutated, the DNA libraries were analyzed by Sanger sequencing with primers listed in Table S2.

### Screening 22c libraries

VNM13 cells containing pBBR1MCS2-GBSV were electroporated with 1.5 μl of the 22c library. Bacteria were plated on LB 20 μg/ml kanamycin and 100 μg/ml ampicillin and incubated overnight at 37 °C. Screening was performed in 2 ml 96-well deep-well plates (Thermo Fisher Scientific #12566612) by inoculating 90 clones from a single 22c library into 500 μl Terrific Broth (TB; BD Difco #243820) containing 20 μg/ml kanamycin and 100 μg/ml ampicillin. Depending on the screening round, three clones of the parental PglS strain (pVNM245 or pVNM306) were used as a positive control. To control for background, three clones of pVNM322, which contains a histidine to alanine substitution at position 324 of PglS to inactivate the protein, were used as negative controls. Plates were sealed with Breathe-Easy semipermeable membranes (Diversified Biotech #Z380059) and were grown overnight at 37 °C at 300 RPM. Stationary phase cultures were then diluted 1:60 into 1.2 mL TB with 20 μg/ml kanamycin and 100 μg/ml ampicillin and cultured for 4 h at 37 °C at 300 RPM. Once cultures reached mid-logarithmic growth, the expression of the GBSV glycan, PglS, and EPA were induced by adding IPTG (Gold Biotechnology #I2481C100) to a final concentration of 0.2 mM. Cells were cultured overnight at 30 °C at 200 RPM.

To extract the periplasm, 1 ml of overnight culture was transferred to a fresh 1 ml 96-well plate (Thermo Fisher Scientific #12566611) and the plate centrifuged for 15 min at 4000*g* at 4 °C. The supernatant was removed *via* aspiration and the pellets resuspended in 200 μl cold 30 mM Tris–HCl pH 8.0, 20% w/v sucrose, 1 mM EDTA, 1 mg/ml lysozyme, and 1 tablet of proteinase inhibitors (Pierce A32953). Cells were incubated without agitation for 1 h at 4 °C. Plates were then centrifuged for 15 min at 4000*g* at 4 °C and 100 μl supernatant removed for ELISA analysis.

## Capture ELISA

Periplasmic sample extracts were analyzed by sandwich ELISA using clear, polystyrene 96-well flat-bottom immunoGrade plates with high-capacity binding (Millipore Sigma, BRAND #781722). Wells were coated overnight at 4 °C without shaking with 100 μl goat anti-EPA antibody diluted to 10 μg/ml in cold 68.6 mM NaHCO_3_, 31.1 mM Na_2_CO_3_. Plates were washed three times with 200 μl 1X PBS, 0.05% Tween-20. Wells were blocked for 2 to 3 h at room temperature in 1% bovine serum albumin (BSA) in 1X PBS. Plates were washed three times with 200 μl 1X PBS, 0.05% Tween-20 and then 100 μl of the periplasmic extracts were bound to the plates by shaking overnight at 250 RPM at 4 °C. Plates were washed three times with 200 μl 1X PBS, 0.05% Tween-20, and 100 μl of the primary antibody, rabbit anti-GBSV, was diluted to 0.1 μg/ml in 1% BSA, 1X PBS, 0.1% Tween-20. Plates were incubated at room temperature for 1 h while shaking at 250 RPM. Plates were washed three times with 200 μl 1X PBS, 0.05% Tween-20. For detection, 100 μl goat anti-rabbit horseradish peroxidase-linked IgG (Invitrogen #31460) diluted to 5 to 80 μg/ml in 1% BSA, 1X PBS, 0.1% Tween-20 was added, and the plates incubated at room temperature for 1 h while shaking at 250 RPM. Plates were washed three times with 200 μl 1X PBS, 0.05% Tween-20, and 100 μl of the 1-Step TMB Substrate Kit (Thermo Fisher Scientific #34021) was added. Plates were developed while shaking at 250 RPM for 15 min at room temperature. Reactions were stopped by adding 100 μl 0.2 to 0.4 M sulfuric acid and the plates read at Absorbance_450_ nm.

For assays comparing the PglS variants with the EPA2 and EPA6 carrier proteins, ELISAs were performed as stated above, except the periplasm was extracted for 1 h at 4 °C in 200 μl cold 175 mM NaCl, 5 mM EDTA, 50 mM Tris–HCL, pH 7.5, with 0.5 mg/ml polymyxin B. Additionally, plates were blocked in 5% BSA in 1 X PBS, and 0.1% Tween-20 for 2 h at room temperature.

### Cloning combinatorial amino acid substitutions in PglS

To generate the combinatorial PglS variants, fragments of *pglS* containing the selected mutations were synthesized as dsDNA synthetic blocks from IDT using their eBlock platform. The eBlock fragments were then assembled into the backbone pVNM306 *via* a Gibson assembly strategy. Briefly, pVNM306 was PCR amplified, *DpnI* treated, and gel purified as described above. The pVNM306 PCR products were amplified such that the region containing the selected residues for mutation were absent, thereby allowing replacement *via* Gibson assembly with the eBlock fragment containing the mutations. pVNM306 was diluted to 10 ng/μl in water and 2 μl was added to every well of a 0.2 ml 96-well semi-skirted plate (Thermo Fisher Scientific #AB-0900) containing 4 μl/well HiFi DNA Assembly Master Mix (New England BioLabs #E2621X). Two microliters of the eBlock fragments at 20 ng/μl (IDT) were added to the vector/ligation mixture. The plate was sealed with sterile foil seals (Excel Scientific AlumaSeal II #AFS-25) and then incubated for 1 h at 50 °C in a PCR machine.

To incorporate N28W and S30F substitutions into the combinatorial library backbones, the single site changes were first constructed into pVNM306. The primer sets N28W Fwd/N28W Rev and S30F Fwd/S30 Rev were used to amplify pVNM306 (Table S2). PCR products were treated with *DpnI* for 2 h at 37 °C to remove template DNA. Gel purified products pVNM306_N28W and pVNM306_S30F were then assembled using NEBuilder HiFi DNA Assembly master mix as described in the manufacturer’s protocol. Two microliters of the HiFi reaction was transformed into 50 μl of chemically competent Stellar competent cells (Takara Bio #ST0213). Transformants were selected on LB supplemented with 100 μg/ml ampicillin, the plasmids isolated (GeneJET Plasmid Miniprep Kit, Thermo Fisher Scientific #K0503), and the clones verified by Sanger sequencing (Genewiz). The resulting strains, pVNM306_N28W and pVNM306_S30F, were then used to construct the combinatorial PglS variants.

All steps for transformation into *E. coli* were performed in a PCR machine. Briefly, 2 μl of the ligation reaction was added with a multichannel to 20 μl Stellar competent cells (Takara Bio #ST0213) in a 96-well semi-skirted plate. The cells were incubated for 30 min at 4 °C, heat shocked for 45 s at 42 °C, and chilled at 4 °C for 2 min. Bacteria were recovered in 80 μl/well super optimal broth (BD Difco #244310) and incubated for 1 h at 37 °C. Cells were plated on LB agar containing 100 μg/ml ampicillin in 6-well plates. Bacteria were incubated overnight at 37 °C.

To validate transformants, a single colony from the overnight incubation was inoculated into 1.2 ml TB (BD Difco #243820) containing 150 μg/ml ampicillin in deep, 2-ml 96-well plates (Thermo Fisher Scientific #12566612). Plates were sealed with Breathe-Easy semipermeable membranes (Diversified Biotech #Z380059) and were grown overnight at 37 °C at 300 RPM. Bacterial cells were pelleted for 15 min at 4100*g* and the supernatant removed by aspiration. Plasmids were then extracted with a PureLink HQ 96 Plasmid Purification Kit per the manufacturer’s instructions (Invitrogen #K210096). To verify the residues were successfully mutated, the DNA libraries were analyzed by Sanger sequencing (Genewiz).

### EPA6-GlcNAc purifications

Electrocompetent *E. coli* VNM45 cells were transformed with plasmids containing EPA6-6xHis-PglS (pVNM599), EPA6-6xHis-PglS_T268F K312E_ (pVNM432), or EPA6-6xHis-PglS_N28W T268F K312E_ (pVNM457) and plated on L-agar containing ampicillin (100 μg/ml). After incubating the plates at 37 °C overnight, five colonies of each strain were used to inoculate 1 L flasks containing 500 ml TB (Corning 46-055-CM) with ampicillin (100 μg/ml). Cultures were grown for 22 h at 30 °C and 200 RPM. Overnight cultures were back diluted to an optical density at 600 nm of 0.08 in six 2 L flasks each containing 1 L TB supplemented ampicillin and incubated at 30 °C and 175 RPM until mid-log (OD 0.4–0.6). At mid-log, all cultures were induced with 0.1 mM IPTG. After 20 h, cells were pelleted *via* centrifugation. The supernatant was discarded, and cell pellets were stored at −20 °C. Frozen cell pellets were resuspended in 100 ml lysis buffer (500 mM NaCl, 20 mM Tris–HCl pH 8.0, 10 mM imidazole) per 1000 OD units with protease inhibitor tablets. The resuspension was strained through cheese cloth and run through a cell disruptor twice at 35,000 psi. The lysate was clarified *via* centrifugation at 18,000*g* for 45 min and passed over 14 ml Nickel NTA agarose beads (Goldbio H-350-500). The column was then washed with 100 ml lysis buffer and 35 ml elution buffer (20 mM Tris–HCl pH 8.0, 300 mM imidazole). The eluate was diafiltrated with Amicon Ultra Centrifugal Filters (UFC9050) into 20 mM Tris–HCl pH 8.0. Once the conductance dropped under 4 mS/cm, the sample was filtered with 0.22 μm Millex-GP PES Filters (SLGP033R) and loaded onto a HiScale column containing Source 15Q resin (Cytiva 17094702). The column was washed with combinations of buffer A (20 mM Tris–HCl pH 8.0) and buffer B (20 mM Tris–HCl pH 8.0, 1 M NaCl). Glycosylated EPA6 proteins were eluted with four column volumes at 20% buffer B as confirmed with SDS-PAGE analysis. These fractions were then pooled, concentrated with Amicon filters, buffer exchanged into Tris-buffered saline, and loaded onto a HiLoad Superdex 200 pg size exclusion chromatography column (Cytiva 28989335). After the conjugates were eluted into Tris-buffered saline, fractions were analyzed with SDS-PAGE, pooled, and confirmed *via* Western blot analysis. Protein concentration of each bioconjugate was determined with a Pierce BCA Protein Assay (Thermo Fisher Scientific 23225), and 40 μg of each was then frozen with dry ice and lyophilized for shipment.

### LC-MS protein digests

Purified glycoproteins were prepared for analysis using in-solution digestion. Briefly, 10 μg of each sample was resuspended in 20% acetonitrile, reduced and alkylated with the addition of 10 mM dithiothreitol for 1 h at room temperature, followed by the addition of 40 mM iodoacetamide for 1 h in the dark. Residual iodoacetamide was quenched with the addition of 40 mM dithiothreitol for 15 min at room temperature. Following reduction and alkylation, samples were diluted 10-fold with 100 mM tetraethylammonium bromide pH 8.5 (Sigma) and digested overnight with Trypsin/Lys-C (1/10 w/w Promega) at 37 °C with shaking at 1000 RPM. Digestions were acidified with formic acid to 0.2% and then polished using C18 Stage tips (Empore C18, Sigma) to ensure the removal of particulate matter. Eluted C18 cleaned up peptide mixtures were dried by vacuum centrifugation at room temperature and stored at −20 °C.

### Glycopeptide LC-MS analysis

Digested glycoproteins were re-suspended in buffer A∗ (2% acetonitrile, 0.1% TFA) and separated using a two-column chromatography set-up composed of a PepMap100 C18 20 mm × 75 μm trap and a PepMap C18 500 mm × 75 μm analytical column (Thermo Fisher Scientific). Samples were concentrated onto the trap column at 5 μl/min for 5 min with buffer A (0.1% formic acid, 2% dimethyl sulfoxide) and then infused into an Orbitrap Fusion Lumos Tribrid Mass Spectrometer (Thermo Fisher Scientific) at 300 nl/min *via* the analytical column using a Dionex Ultimate 3000 UPLC (Thermo Fisher Scientific). Ninety-six minute analytical runs were undertaken by altering the buffer composition from 2% buffer B (0.1% formic acid, 77.9% acetonitrile, 2% dimethyl sulfoxide) to 28% B over 66 min, then from 22% B to 45% B over 10 min, and then from 45% B to 80% B over 2 min. The composition was held at 80% B for 3 min and then dropped to 2% B over 5 min before being held at 2% B for another 10 min. The Lumos Mass Spectrometer was operated in a data-dependent mode automatically switching between the acquisition of a single Orbitrap MS scan (maximal injection time of 246 ms, an automated gain control (AGC) set to 100% and a resolution of 120 k) every 3 s and three Orbitrap tandem mass spectrometry scans of each ion; a stepped collision energy high-energy collision dissociation (HCD) scan (using 15%, 30% and 45% normalized collision energy Stepping, maximal injection time of 250 ms, an AGC set to a maximum of 500% and a resolution of 30 k), an EThcD scan (normalized collision energy 15%, maximal injection time of 250 ms, AGC set to 500%, a resolution of 30 k, and using the extended mass range setting to improve the detection of high mass glycopeptide fragment ions); and a collision-induced dissociation scan (maximal injection time of 250 ms, an AGC set to a maximum of 500% and a resolution of 30 k). To confirm the absence of doubly glycosylated glycoforms of glycosylated EPA purified from cells expressing the WT PglS, parallel reaction monitoring using both HCD and EThcD analyses were undertaken assessing the doubly, singly, and unmodified *m/z* of the EPA6 glycopeptides.

### Glycopeptide identifications

Glycopeptide analysis was undertaken using MSFragger-Glyco within MSFragger (version 22.0). Samples were searched with a Tryptic specificity allowing a maximum of two missed cleavage events and carbamidomethyl set as a fixed modification of cysteine, while oxidation of methionine and glycosylation of serine with HexNAc (204.0866 Da) were allowed as variable modifications. The maximum size of considered peptides was increased from 5000 to 7000 and the maximum amino acid length of peptides increased to 70 amino acids to allow the monitoring of large EPA glycopeptides. For HCD searches y, b, Y, y∼/b∼ (y and b ion with the addition of HexNAc) were allowed. A maximum mass precursor tolerance of 20 ppm was allowed at both the MS1 and MS2 levels. Samples were searched against a custom database containing the EPA6 sequence at a 1% false discovery rate. To ensure high data quality assigned glycopeptides were manually assessed and the HCD and EThcD spectra assigned to each unique glycopeptide annotated with the Interactive Peptide Spectral Annotator (http://www.interactivepeptidespectralannotator.com/PeptideAnnotator.html). Extracted ion chromatograms of unmodified peptides and glycoforms of interest were manually extracted and quantification undertaken using the area under the curve of the extracted features within Freestyle (1.7 SP Thermo Fisher Scientific).

## Western blotting

VNM13 was electroporated with pVNM432, pVNM457, pVNM599, pVNM771, pVNM772, or pVNM773, separately. Two single colonies were picked from each plate and grown in 5 ml Corning TB. The cells were incubated at 30 °C with 200 RPM until mid-logarithmic phase was reached, at which point 100 μM IPTG was added to induce the expression of EPA6. Cultures were incubated at 30 °C with 200 RPM overnight. One unit of OD_600_ of cells (1 OD_600_ unit/ml) was harvested for each culture. Bacteria were pelleted and suspended in 200 μl 2 × Laemmli buffer which was supplemented with 5% 2-mercaptoethanol. The samples were incubated at 100 °C for 10 min. Five microliters samples were loaded in each well of a 7.5% TGX Bio-Rad SDS-PAGE gel. After transferring to nitrocellulose membrane, the membrane was blocked in blocking buffer (LICOR Intercept TBS). Rabbit anti-EPA (MilliporeSigma catalog #P2318) and mouse anti-RNA polymerase beta subunit (BioLegend catalog # 663905) were used to detect the expression of EPA and the internal standard RNA polymerase beta subunit, respectively. IRDye 680RD Goat anti-Rabbit IgG Secondary Antibody (LICOR catalog # 926-68071) and IRDye 800CW Goat anti-Mouse IgG Secondary Antibody (LICOR catalog # 926-32210) were used as secondary antibodies. The membrane was imaged using a LICOR Odyssey XF imager.

## Data availability

All mass spectrometry data (RAW files and FragPipe outputs) have been deposited into the PRIDE ProteomeXchange repository under the accession PXD060600 and are accessible *via* the reviewer login Username: reviewer_pxd060600@ebi.ac.uk Password: swRLFEwdFNZf.

All data are contained within the article and supporting information.

## Supporting information

This article contains supporting information.

## Conflict of interest

R. L. E., K. N. M., J. C. M, C. J. K., A. J. H., J. J. M., B. Z., L. S. R., and C. M. H. each have financial interests in VaxNewMo, LLC (doing business as Omniose). The other authors declare that they have no conflicts of interest with the contents of this article.
