# Supplementary material for: Enhancing O-linking oligosaccharyltransferase functionality through directed evolution
Source: J Biol Chem. 2025 Nov 5;302(1):110885. doi: 10.1016/j.jbc.2025.110885 (PMC12800693; doi:10.1016/j.jbc.2025.110885)
Supplement: Figure S1 [file mmc1.docx]

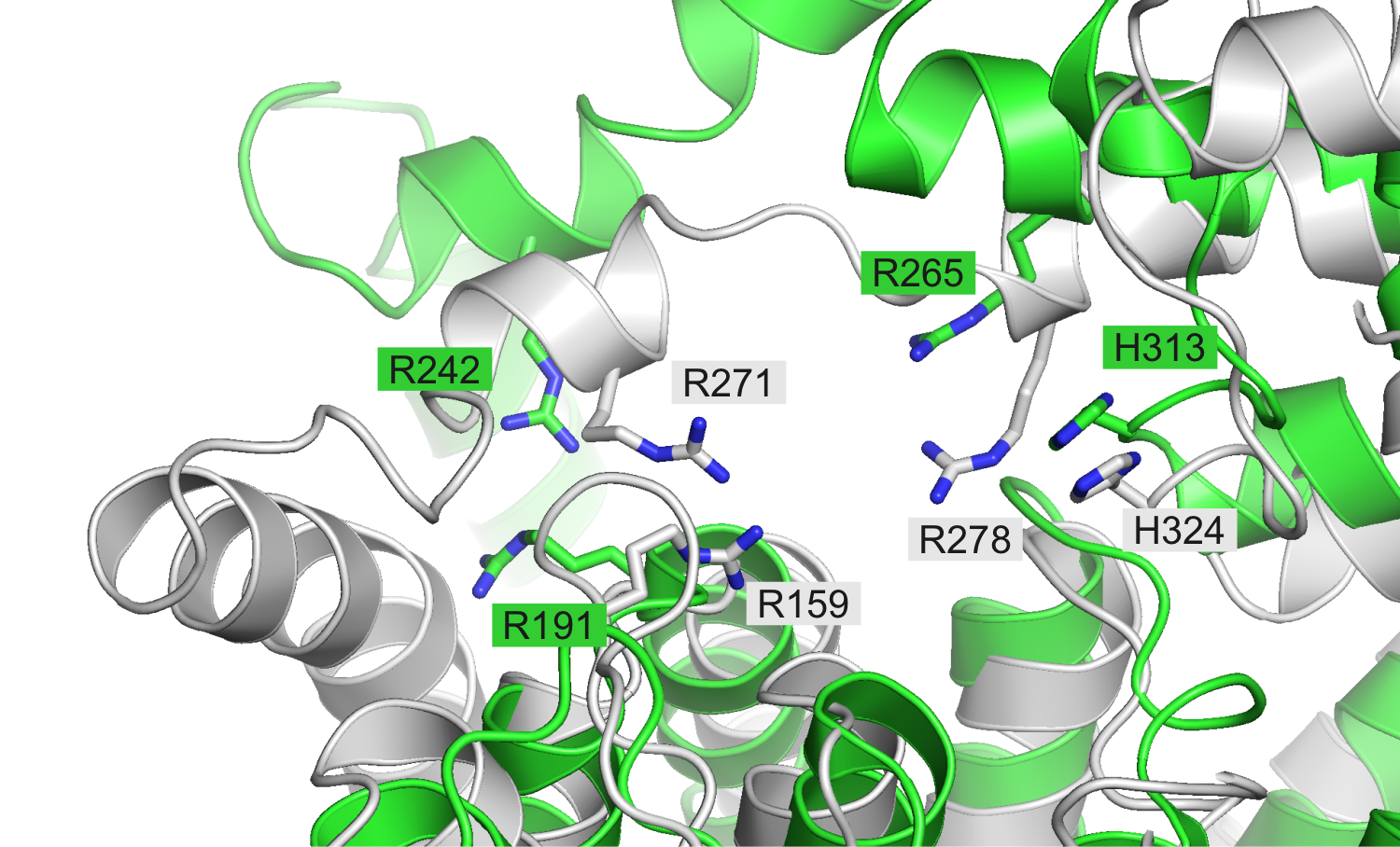


**Figure S1**. Aligned structures of *C. metallidurans* WaaL (PDB ID: 7TPJ, chain A) and the PglS AlphaFold model. PglS is shown in light gray and WaaL in green. Analogous residues are labeled with gray and green backgrounds corresponding to the PglS and WaaL residues, respectively. The RMSD for the structures was 7.4 Å. The alignment was performed using Pymol ver. 2.5.2 (Schrödinger).
