# Supplementary material for: Enhancing O-linking oligosaccharyltransferase functionality through directed evolution
Source: J Biol Chem. 2025 Nov 5;302(1):110885. doi: 10.1016/j.jbc.2025.110885 (PMC12800693; doi:10.1016/j.jbc.2025.110885)
Supplement: Figure S2 [file mmc2.pptx]

## Slide 1
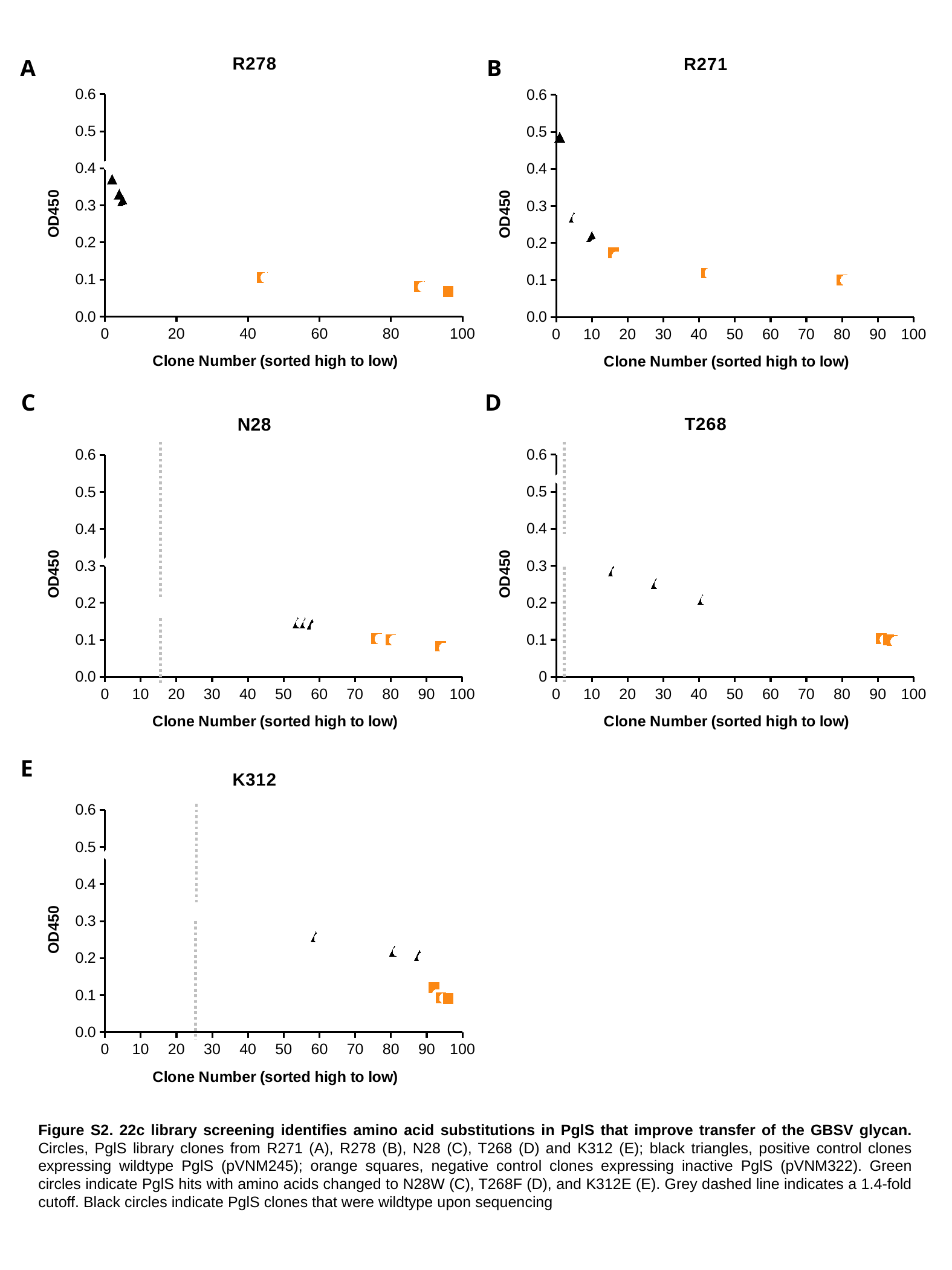

### Chart: R278
| Category | OD450 |
|---|---|
### Chart: R271
| Category | ELISA OD450 |
|---|---|A
B
C
D
### Chart: N28
| Category | OD450 |
|---|---|
### Chart: T268
| Category | ELISA OD450 |
|---|---|E
### Chart: K312
| Category | ELISA OD450 |
|---|---|Figure S2. 22c library screening identifies amino acid substitutions in PglS that improve transfer of the GBSV glycan. Circles, PglS library clones from R271 (A), R278 (B), N28 (C), T268 (D) and K312 (E); black triangles, positive control clones expressing wildtype PglS (pVNM245); orange squares, negative control clones expressing inactive PglS (pVNM322). Green circles indicate PglS hits with amino acids changed to N28W (C), T268F (D), and K312E (E). Grey dashed line indicates a 1.4-fold cutoff. Black circles indicate PglS clones that were wildtype upon sequencing
