# Supplementary material for: Enhancing O-linking oligosaccharyltransferase functionality through directed evolution
Source: J Biol Chem. 2025 Nov 5;302(1):110885. doi: 10.1016/j.jbc.2025.110885 (PMC12800693; doi:10.1016/j.jbc.2025.110885)
Supplement: Figure S3 [file mmc3.pptx]

## Slide 1
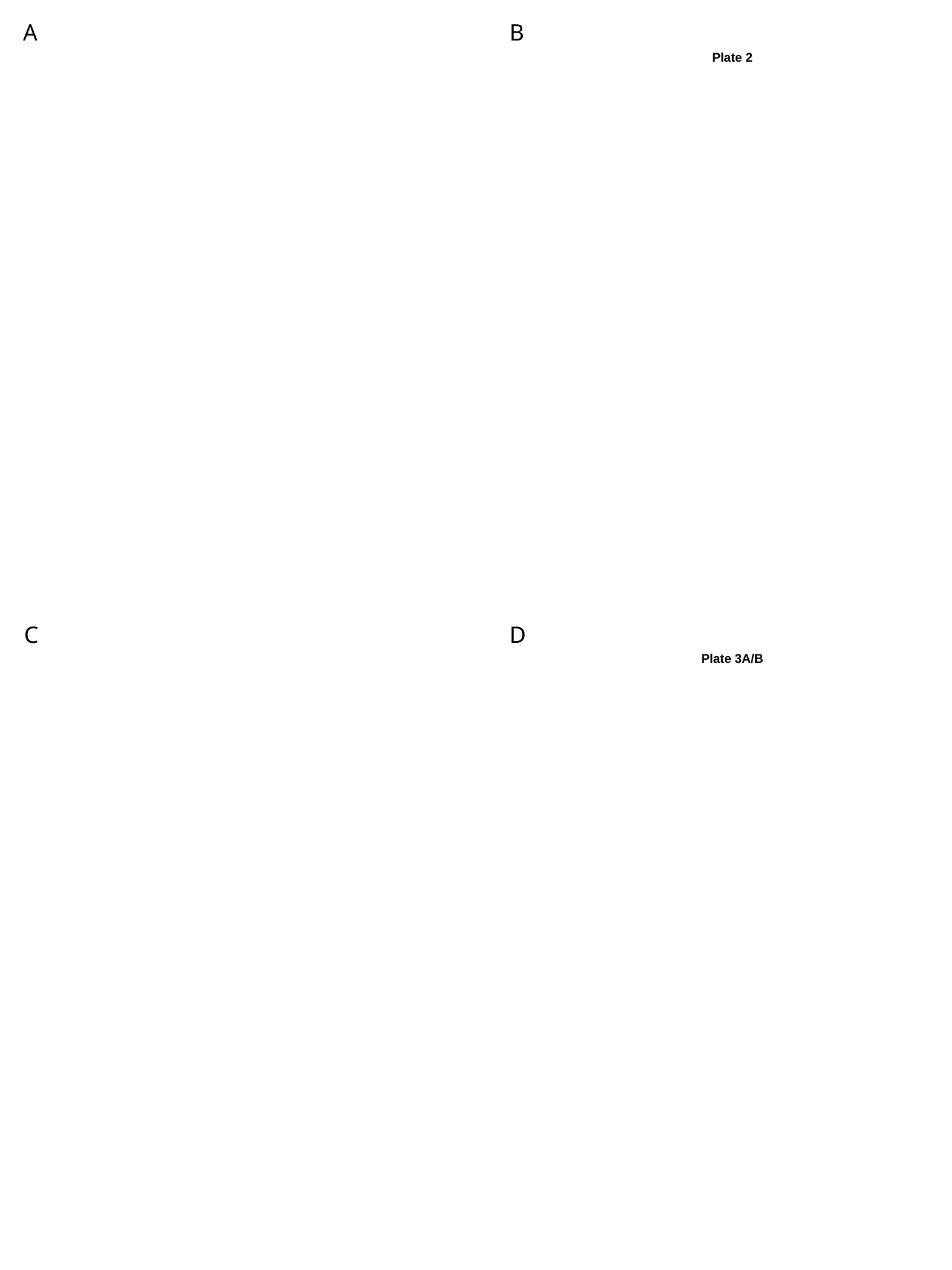

A
B
Plate 2
C
D
Plate 3A/B

## Slide 2
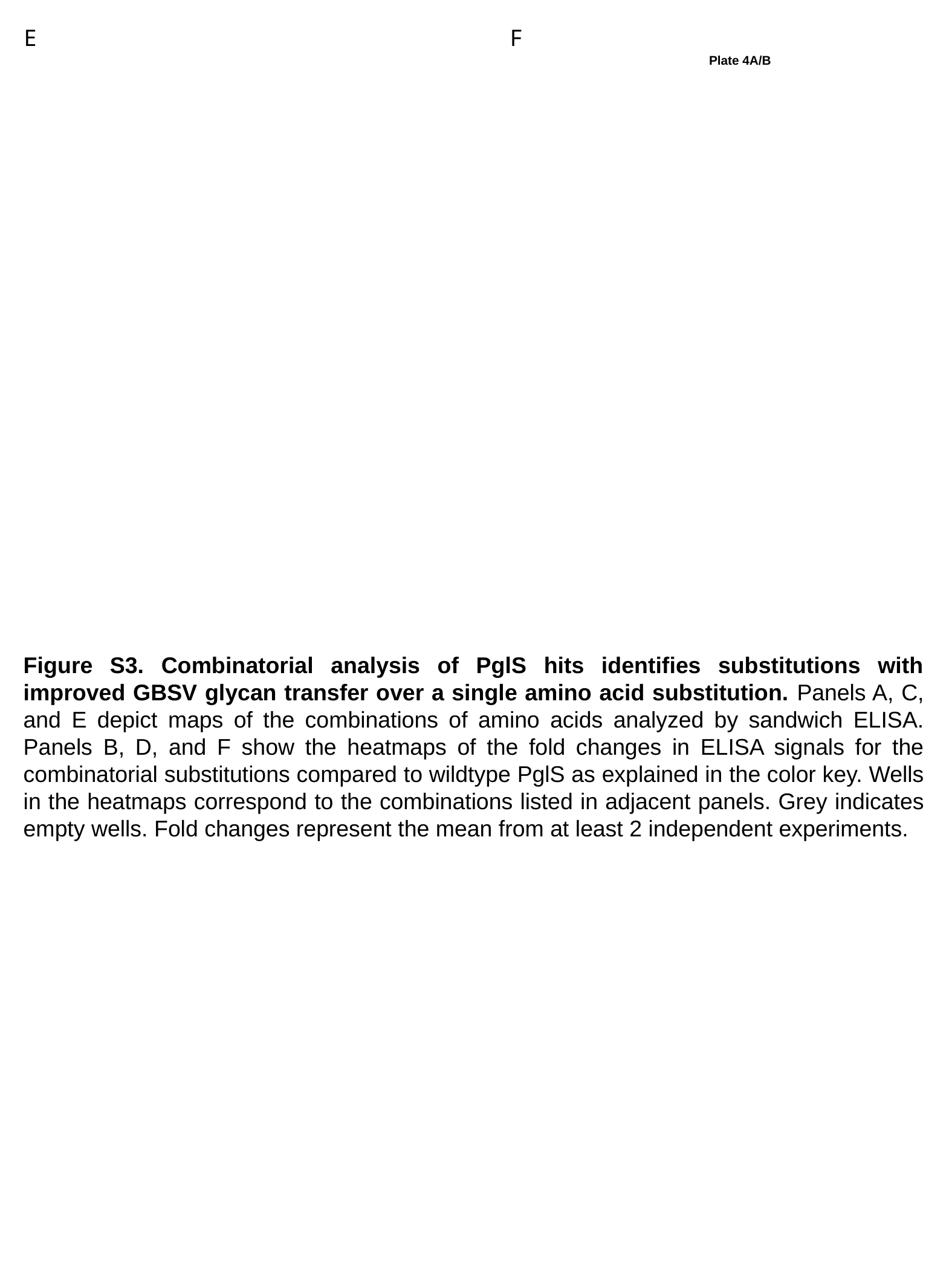

E
F
Plate 4A/B
Figure S3. Combinatorial analysis of PglS hits identifies substitutions with improved GBSV glycan transfer over a single amino acid substitution. Panels A, C, and E depict maps of the combinations of amino acids analyzed by sandwich ELISA. Panels B, D, and F show the heatmaps of the fold changes in ELISA signals for the combinatorial substitutions compared to wildtype PglS as explained in the color key. Wells in the heatmaps correspond to the combinations listed in adjacent panels. Grey indicates empty wells. Fold changes represent the mean from at least 2 independent experiments.
