# Supplementary material for: Enhancing O-linking oligosaccharyltransferase functionality through directed evolution
Source: J Biol Chem. 2025 Nov 5;302(1):110885. doi: 10.1016/j.jbc.2025.110885 (PMC12800693; doi:10.1016/j.jbc.2025.110885)
Supplement: Figure S4 [file mmc4.pptx]

## Slide 1
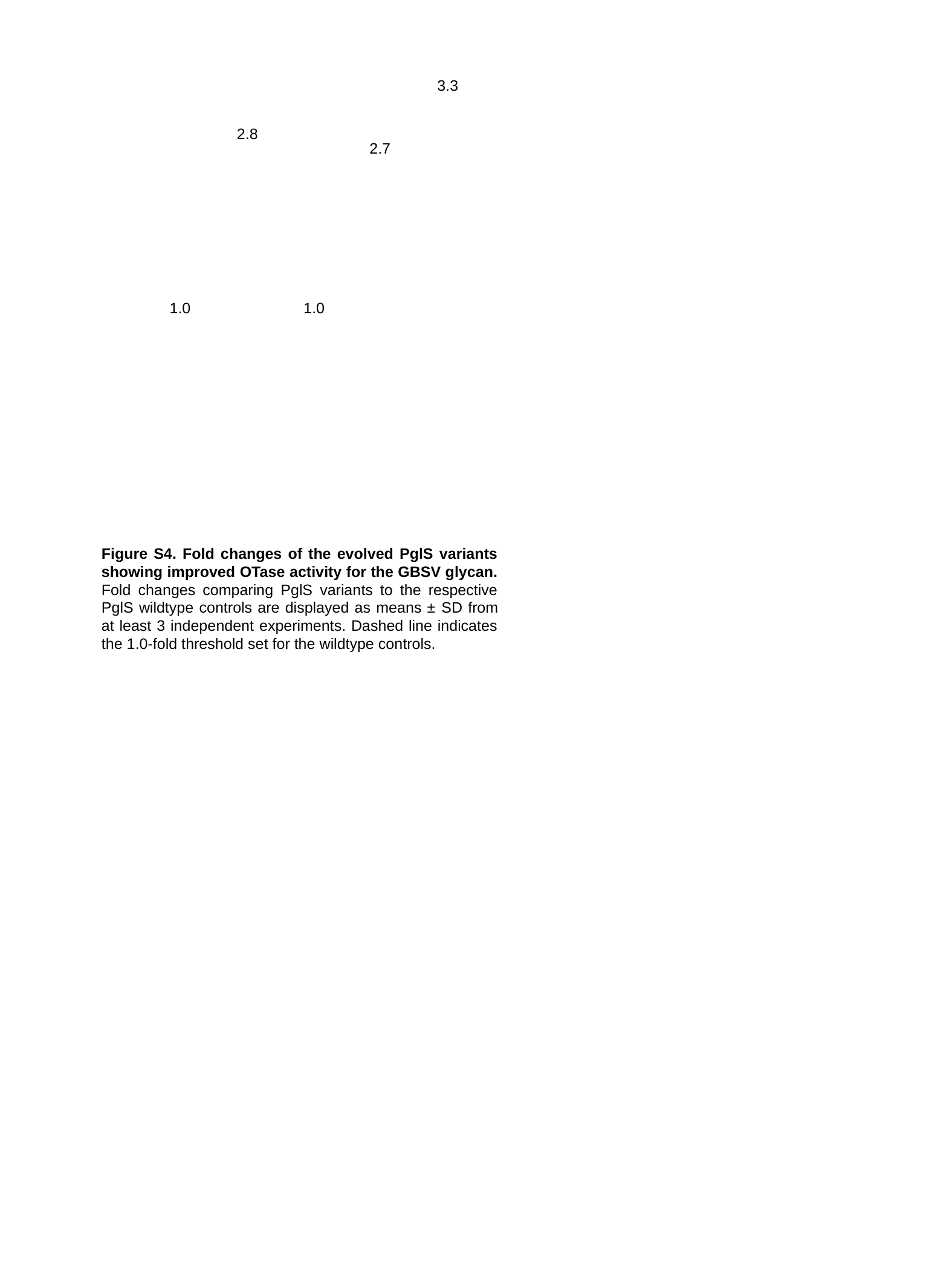

3.3
2.8
2.7
1.0
1.0
Figure S4. Fold changes of the evolved PglS variants showing improved OTase activity for the GBSV glycan. Fold changes comparing PglS variants to the respective PglS wildtype controls are displayed as means ± SD from at least 3 independent experiments. Dashed line indicates the 1.0-fold threshold set for the wildtype controls.
