# Supplementary material for: Enhancing O-linking oligosaccharyltransferase functionality through directed evolution
Source: J Biol Chem. 2025 Nov 5;302(1):110885. doi: 10.1016/j.jbc.2025.110885 (PMC12800693; doi:10.1016/j.jbc.2025.110885)
Supplement: Figure S5 [file mmc5.pptx]

## Slide 1
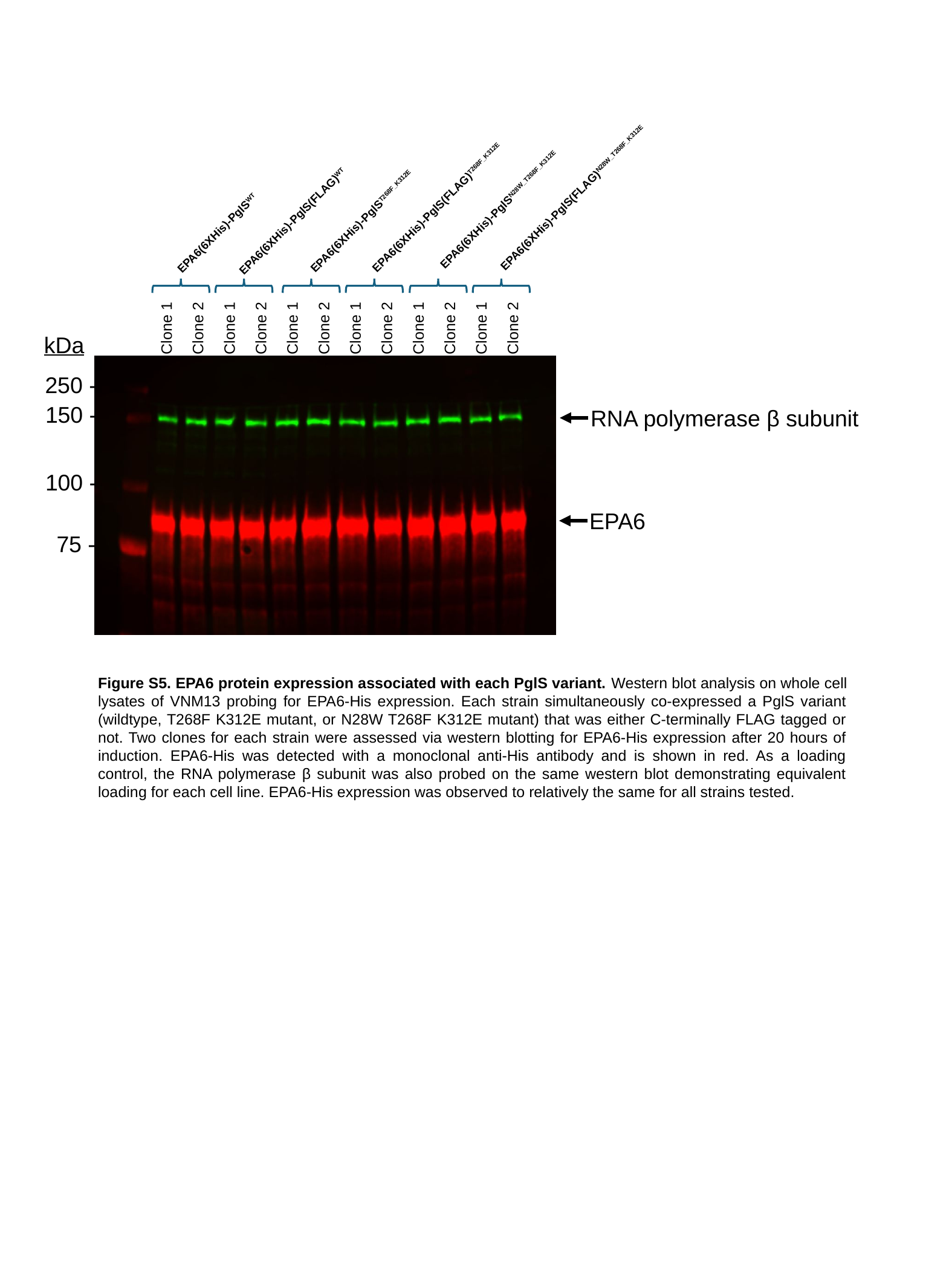

EPA6(6XHis)-PglS(FLAG)N28W_T268F_K312E
EPA6(6XHis)-PglS(FLAG)T268F_K312E
EPA6(6XHis)-PglSN28W_T268F_K312E
EPA6(6XHis)-PglST268F_K312E
EPA6(6XHis)-PglS(FLAG)WT
EPA6(6XHis)-PglSWT
Clone 1
Clone 2
Clone 1
Clone 2
Clone 1
Clone 2
Clone 1
Clone 2
Clone 1
Clone 2
Clone 1
Clone 2
kDa
250 -
150 -
RNA polymerase β subunit
100 -
EPA6
75 -
Figure S5. EPA6 protein expression associated with each PglS variant. Western blot analysis on whole cell lysates of VNM13 probing for EPA6-His expression. Each strain simultaneously co-expressed a PglS variant (wildtype, T268F K312E mutant, or N28W T268F K312E mutant) that was either C-terminally FLAG tagged or not. Two clones for each strain were assessed via western blotting for EPA6-His expression after 20 hours of induction. EPA6-His was detected with a monoclonal anti-His antibody and is shown in red. As a loading control, the RNA polymerase β subunit was also probed on the same western blot demonstrating equivalent loading for each cell line. EPA6-His expression was observed to relatively the same for all strains tested.
