# Supplementary material for: Enhancing O-linking oligosaccharyltransferase functionality through directed evolution
Source: J Biol Chem. 2025 Nov 5;302(1):110885. doi: 10.1016/j.jbc.2025.110885 (PMC12800693; doi:10.1016/j.jbc.2025.110885)
Supplement: Figure S6 [file mmc6.docx]

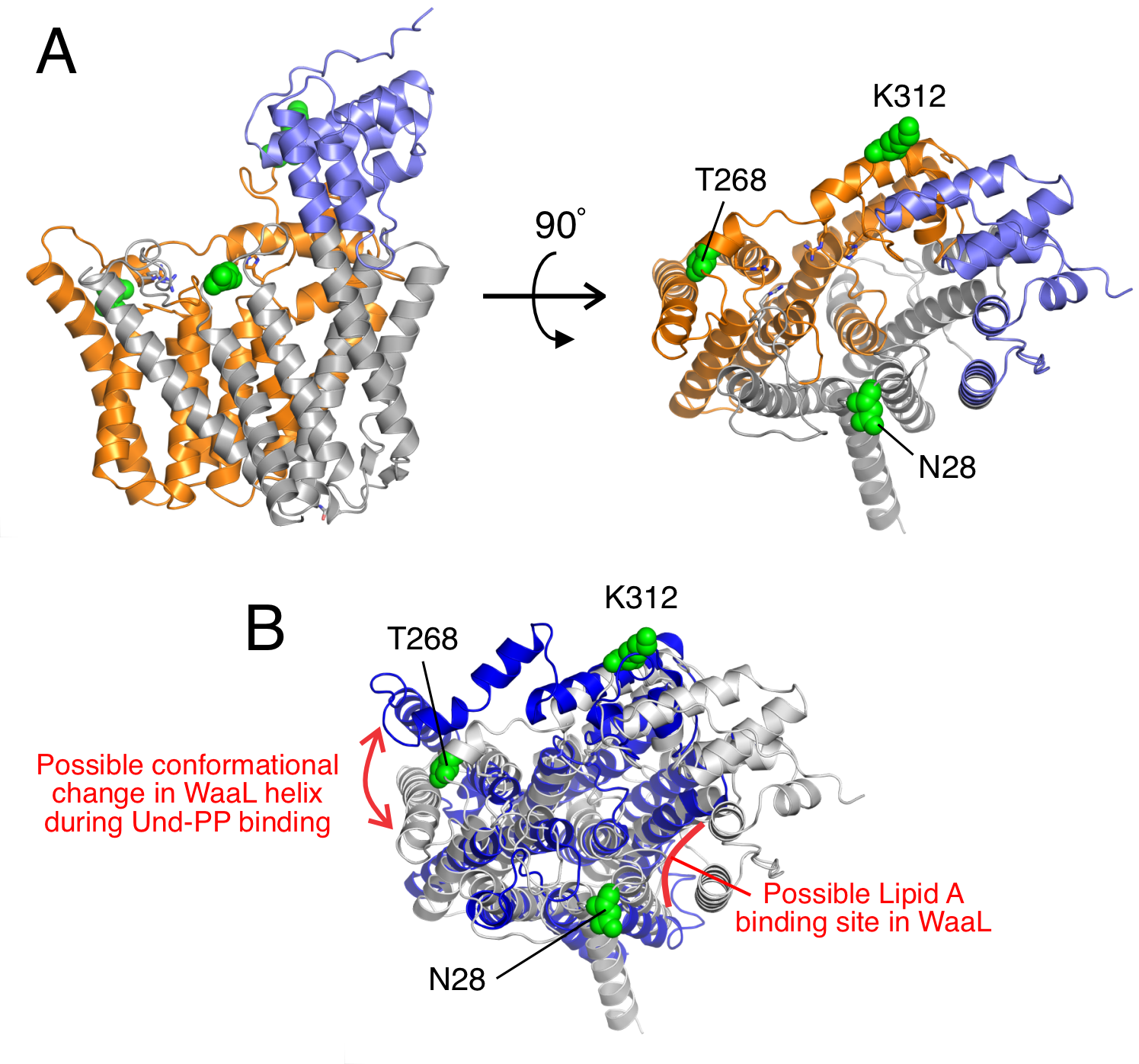


**Figure S6**. AlphaFold structure of PglS from *Acinetobacter baylyi* ADP1 with residues from the PglS triple variant marked. A) Two orientations of the N28 T268 K312 residues mapped onto the *in silico* PglS structure. The RfaL domain is colored orange and the wzy_C domain is colored blue. B) Structural alignment of the Cryo-EM WaaL structure (dark blue) and AlphaFold PglS model (gray). Based on structural homology between PglS and WaaL (rmsd the three residues identified in our screen are positioned near possible regions of WaaL involved in gating glycan donor entry via a dynamic helix or binding of the recipient substrate on the opposite side of WaaL/PglS. The structure alignment was made using Pymol version 3.1.6.1 (Schrödinger) using the extra_fit command.
