# Supplementary material for: Enhancing O-linking oligosaccharyltransferase functionality through directed evolution
Source: J Biol Chem. 2025 Nov 5;302(1):110885. doi: 10.1016/j.jbc.2025.110885 (PMC12800693; doi:10.1016/j.jbc.2025.110885)
Supplement: Supporting Information Table Legends [file mmc7.docx]

**Supporting Information Table Legends**

**Table S1. PglS residues screened by ELISA.** Column A) The complete list of all PglS amino acid residues targeted for mutagenesis and capture ELISA for altered oligosaccharyltransferase activity screening. Residues with an asterisk denote that targeted mutagenesis approaches were employed for screening as the 22- libraries could not be generated for those residues. Column B) Details which *pglS* containing expression vector was used for library generation (22-c or targeted mutagenesis). The pVNM245 and pVNM306 vectors are identical with the exception that the *pglS* gene in pVNM306 is an *E. coli* codon optimized variant.

**Table S2. Primers.** Primer sequences used in this study for 22-c trick library generation (Tab 1) or other cloning primers and sequence validation primers (Tab 2).

**Table S3. eBlock maps and sequences for combinatorial PglS substitutions.** A complete schematic map of the combinations of PglS substitutions tested in this study (Tab 1). Tabs 2-5 show the nucleotide sequence of the eBlock fragment synthesized to obtain a particular combination of substitutions listed in Tab 1.

**Table S4. EPA_Wildtype PglS_PSM.** The Peptide-Spectrum Match (PSM) file for EPA6 peptides and glycopeptides analyzed via tandem MS/MS purified from the *E. coli* W3110 ∆*gtrABS* ∆*wzzE-wecG* mutant over-expressing the wildtype PglS variant.

**Table S5. EPA_PglS T268F_K312E_PSM.** The Peptide-Spectrum Match (PSM) file for EPA6 peptides and glycopeptides analyzed via tandem MS/MS purified from the *E. coli* W3110 ∆*gtrABS* ∆*wzzE-wecG* mutant over-expressing the double PglS_T268F K312E_ variant.

**Table S6. EPA_PglS N28W_T268F_K312E_PSM.** The Peptide-Spectrum Match (PSM) file for EPA6 peptides and glycopeptides analyzed via tandem MS/MS purified from the *E. coli* W3110 ∆*gtrABS* ∆*wzzE-wecG* mutant over-expressing the triple PglS_N28W T268F K312E_ variant.
